# Vasopressin and angiotensin II pathways differentially modulate human fear response dynamics to looming threats

**DOI:** 10.1371/journal.pbio.3003668

**Published:** 2026-02-24

**Authors:** Mengfan Han, Wenyi Dong, Kun Fu, Junjie Wang, Yuanhang Xu, Yueyuan Zheng, Keith Kendrick, Stefania Ferraro, Ting Xu, Dezhong Yao, Benjamin Becker

**Affiliations:** 1 The Center of Psychosomatic Medicine, Sichuan Provincial Center for Mental Health, Sichuan Provincial People’s Hospital, University of Electronic Science and Technology of China, Chengdu, China; 2 MOE Key Laboratory for Neuroinformation, School of Life Science and Technology, University of Electronic Science and Technology of China, Chengdu, China; 3 Division of Social Science, Hong Kong University of Science and Technology, Hong Kong, Hong Kong SAR, China; 4 Faculty of Psychology, Southwest University, Chongqing, China; 5 Department of Psychology, The University of Hong Kong, Hong Kong, Hong Kong SAR, China; 6 Strategic Research Theme: AI, Society & Social Dynamics, Faculty of Social Sciences, The University of Hong Kong, Hong Kong, Hong Kong SAR, China; Universitat Jaume 1, SPAIN

## Abstract

While basal threat processing dynamics (e.g., visual looming) are well characterized in animals, the underlying mechanisms and their modulation by neuropeptide systems with different modulatory roles in threat processing (vasopressin, angiotensin II) remain poorly understood in humans. In a randomized, placebo-controlled eye-tracking study (*N* = 111), we administered vasopressin (AVP) or an angiotensin II receptor blocker (via Losartan, LT) during a time-to-collision threat paradigm. This study was prospectively registered at ClinicalTrials.gov (NCT06329076, NCT06329063) on April 11, 2024, prior to participant enrollment. Behaviorally, AVP induced a systematic time overestimation while LT induced temporal compression and reduced state anxiety. Pupillometry revealed distinguishable profiles: AVP induced sustained constriction during stimulus approach followed by post-stimulus threat-specific dilation, LT maintained sustained pupillary constriction throughout both approach and occlusion phases yet preserving threat-specificity, while placebo (PLC) showed no threat-specific modulation. A computational framework (combining Functional Principal Component Analysis, clustering, and Markov chain analysis) underscored the distinct modulations: AVP stabilized a high-arousal state characterized by the co-activation of vigilance, threat-proactive preparation and a shift from perception to internal simulation. LT suppressed transitions to high-arousal states and exhibited maximal sequence entropy, reflecting flexible response patterns—contrasting with placebo’s lowest entropy dynamics. These results demonstrate that AVP and LT differentially regulate basal threat processing via separable neuropeptide pathways: AVP sustains hypervigilance while LT promotes anxiolysis and adaptive flexibility. Our findings suggest neuropeptide pathway-specific targets maladaptive threat processing in trauma- or anxiety-related disorders.

## Abstract 2

Neuromodulatory pathways are involved in threat-processing, but the roles of specific neuropeptides in the human threat response are not clear. This study in humans characterized the distinct impacts of pharmacological manipulation of two neuropeptide pathways on responses to looming stimuli, with implications for understanding trauma- or anxiety-related disorders.

## Introduction

The subjective experience of emotions varies across individuals and requires the coordinated involvement of distributed cortical and subcortical systems [1–3]. Some basic forms of “primitive” affective mechanisms such as defensive reactions to rapidly approaching objects (“looming”) appear to be innate, highly preserved across phylogenetic and ontogenetic evolution and tightly linked to specific (subcortical) circuits [4,5]. The cognitive and neural bases of the corresponding affective processes have been extensively studied within broader models of emotion. Foundational work has highlighted the central role of subcortical structures, including the amygdala, in the rapid and automatic detection of motivationally salient stimuli, particularly threats [6,7]. This rapid threat detection system interacts with cortical networks to generate conscious emotional experiences and coordinate adaptive behavioral responses [8].

In interaction with these foundational mechanisms, the visual system can rapidly detect impending—and potentially threatening—collisions via estimating the accelerating expansion of objects on the retina (looming). Looming presents a fundamental, evolutionary, and highly conserved threat detection mechanisms, which can be observed from fruit flies to humans [9,10]. This innate perceptual sensitivity to approaching stimuli that specifies the time-to-collision independent of size or distance enables avoiding collisions and rapid defensive responses [11–13].

Although looming perception has been traditionally viewed as a basic visual computation, recent studies have demonstrated that looming is modulated by the semantic and affective content of the stimuli, such that threatening stimuli are typically underestimated in the time to collision by 20%–30%, a time compression effect that may critically facilitate faster defensive reactions [13–15]. However, in natural environments, threatening objects such as predators often temporarily disappear from view, raising critical questions about how threat processing mechanisms operate without continuous visual input and how imagination maintains threat assessment during such periods. Understanding these mechanisms is crucial for developing ecologically valid models of threat processing and may help to determine how these processes can be targeted in mental disorders characterized by excessive threat processing.

The neuropeptide systems arginine vasopressin (AVP) and renin-angiotensin (RAS) have been associated with threat-related processing across species. AVP, synthesized in the hypothalamus, enhances threat processing by increasing the extended amygdala sensitivity to threat information and modulating prefrontal-amygdala circuits [16,17], with pronounced effects in males [18]. The RAS, in particular blockade of the RAS II AT1 receptors, has been associated with decreased fear by reducing amygdala reactivity to threat and enhancing prefrontal regulatory control [5,19–21]. While both systems influence threat processing through partially overlapping neural circuits and show complex synergistic effects in regulating physiological indices of threat responsivity including blood pressure and stress responses [22–24] their respective roles in looming threat processing remain to be explored.

Pupillary responses offer unique advantages as precise and non-invasive indicators of threat processing dynamics [25–27]. Through tracking the activity of the locus coeruleus-norepinephrine (LC-NE) system pupil dilation provides high temporal resolution information about arousal related to cognitive and affective engagement [28,29], As such, pupil responses can reveal the continuous temporal dynamics of threat processing.

The LC-NE arousal system has shown a high sensitivity to both AVP [30] and RAS [31] modulation which render pupillary responses an ideal indicator for investigating the neurobiological basis of their modulatory role on threat processing.

Against this background we aimed at determining the role of two neuropeptide systems (AVP, RAS) in basal threat-related mechanisms, in particular to determine their effects on threat processing during looming via determining the effects of single dosages of both, AVP and a selective and competitive angiotensin receptor II antagonists (Losartan, LT) on behavioral and pupillary responses during the looming fear paradigm in combination with computational analytic approaches. This novel combination allows us to dissect the distinct contributions of these systems to threat processing and explore potential therapeutic implications. The hypothesized effects were tested in a pre-registered randomized placebo-controlled double-blind psychopharmacological eye tracking design during which participants were administered oral AVP, LT, or placebo (PLC).

To provide an accurate test of the complex hypotheses, we established a novel expansion of the threat looming paradigm and established a comprehensive analytic framework. This expansion integrates both visual and imagination components, comprising two phases: a one-second “visual stimulus amplification” phase providing initial approach velocity information, followed by an “imagined stimulus approach” phase where the visual threat stimulus disappears after the approach information has been acquired. This design builds on substantial evidence that threat-related neural systems remain active even without direct visual input [8,32], and amygdala-centered defensive circuits continue processing threats when visual stimuli are subliminal or masked [33,34], indicating robust threat maintenance mechanisms during temporary visual occlusion.

To characterize the complex dynamics of threat processing, we developed an integrated analytical framework combining pupillary features, behavioral responses, and temporal measurements. This multivariate approach identifies distinct threat response patterns and their pharmacological modulation through dimensional reduction and clustering techniques. By simultaneously analyzing multiple pupillary characteristics and behavioral measures, we reveal how different pharmacological treatments shape overall threat processing strategies. This systematic approach advances beyond traditional univariate analyses to capture the temporal evolution and multidimensional nature of threat responses under different pharmacological conditions.

Our specific aims in this study were to: (1) characterize threat processing dynamics during looming through pupillary responses, (2) examine the maintenance of threat perception during visual occlusion, (3) investigate pharmacological modulation of the identified threat processes, and (4) identify distinct response patterns through Functional Principal Component Analysis (FPCA)–clustering–Markov chain analysis. The findings may reveal innovative targets for testing novel interventions for disorders characterized by elevated threat reactivity and have important implications for how neuropeptides shape defensive behaviors.

## Methods

### 1. Ethics statement

The study was approved by the local ethics committee of the University of Electronic Science and Technology of China (UESTC) and conducted according to the Declaration of Helsinki. The study was pre-registered (ClinicalTrials.gov ID: https://clinicaltrials.gov/ct2/show/NCT06329063, https://clinicaltrials.gov/ct2/show/NCT06329076) on March 15, 2024, following the establishment of technical implementation starting on March 5, 2024. All participants provided written informed consent prior to their inclusion in the study and received monetary compensation (140 RMB).

### 2. Participants and study design

**Fig 1:**
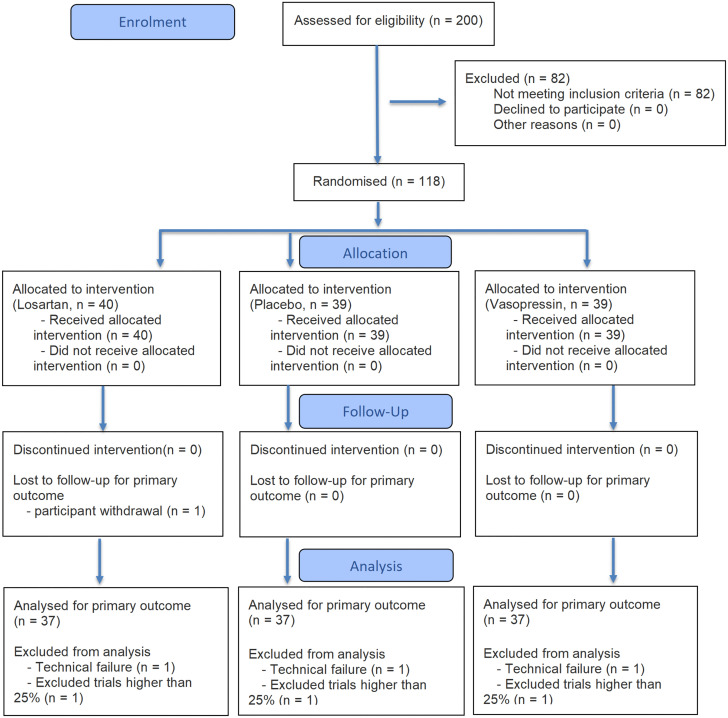
Consolidated standards of reporting trials (CONSORT) flow chart. Detailed exclusion criteria are provided in the S1 Text.

Following screening against the recruitment criteria, a final sample of 111 healthy volunteers (*n* = 37 per treatment group) was included in the analysis (see Consolidated standards of reporting trials (CONSORT) flowchart, Fig 1). This sample size exceeds the a priori requirement of 78 (determined by G*Power for a Treatment (3) × Sex (2) × IsThreaten (2) × Physical Stimulus Velocity (5 levels, from very slow to very fast; see Section 3.3 for detailed classification) mixed-design ANOVA; *f* = 0.20, *α* = 0.05, power = 0.95) to ensure robust group matching and account for potential exclusions.

Eligible participants had normal or corrected-to-normal vision, no history of psychiatric or neurological disorders, and no recent use of any medication or substances (see Supporting information (S1 Text) for full exclusion criteria).

In a randomized, double-blind, placebo-controlled pharmacological behavioral and eye-tracking design (for details on randomization and blinding, see Supporting information (S1 Text)), participants were randomly assigned to receive either AVP, LT, or PLC (mean age ± SD: 21.90 ± 2.31 years; no significant age differences between groups, *F*(2,108) = 0.55, *p* = .58). The trial employed a parallel-group design with a 1:1:1 allocation ratio and was exploratory in nature.

### 3. Experimental procedures

#### 3.1 Baseline assessments.

**Fig 2:**
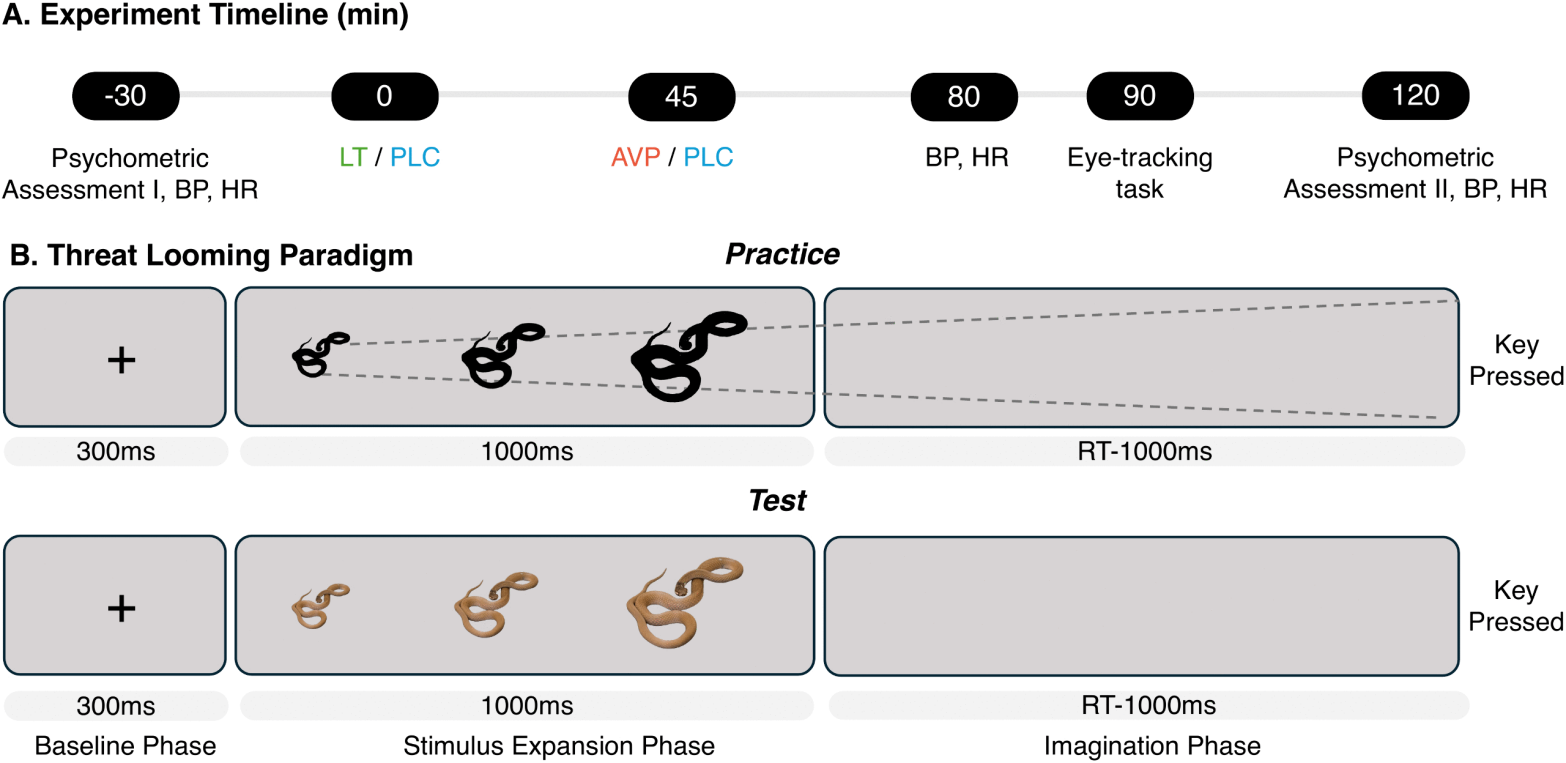
Experimental timeline and task structure. **(A)** Sequence of drug administration and behavioral testing. **(B)** Example trial of the threat looming task. **Image Credits:** The snake photograph is by chris_barnesoz (Wikimedia Commons, CC BY 4.0).

Prior to drug administration, participants completed validated Chinese versions of standardized questionnaires assessing affective states and fear-related traits, including the Positive and Negative Affect Schedule (PANAS) [35]; State-Trait Anxiety Inventory (STAI) [36]; Liebowitz Social Anxiety Scale (LSAS) [37]; and Animal Fear Questionnaire (AFQ) [38]. Blood pressure and heart rate were monitored at three time points: pre-administration, peak drug effect (45 min post-administration for AVP group, 90 min post-administration for LT group), and post-experiment (similar in [21,39], Fig 2A) to control for unspecific cardiovascular effects of the treatments.

#### 3.2 Drug administration.

Treatment doses were selected based on previous research demonstrating efficacy in modulating emotional processing including fear-related processes: 20 IU AVP [39] and 50 mg LT [19–21].

We employed a novel spray-based oral delivery method for AVP [40] to prevent direct entry into the olfactory system and better isolate peripheral effects [41]. Following our validated protocol, participants received six alternating 0.1 ml puffs (total volume 0.6 ml, Supplementary Method (S1 Text)). Placebo sprays were identical in composition but without AVP. LT and its placebo were administered in visually matched capsules containing either 50 mg LT or no active ingredient.

To ensure effects of both treatments during peak plasma windows (based on established pharmacokinetic profiles showing peak effects at 45 min for AVP and 90 min for LT) and maintain double-blinding during tasks, we implemented a two-stage administration protocol:

- Group 1: 50 mg LT capsule (90 min pre-task) + PLC spray (45 min pre-task)
- Group 2: PLC capsule (90 min pre-task) + PLC spray (45 min pre-task)
- Group 3: PLC capsule (90 min pre-task) + 20 IU AVP spray (45 min pre-task)

All treatments were administered by research staff blind to treatment conditions. Successful blinding was confirmed, as participants’ post-experiment treatment guesses did not exceed chance level (*χ*²(2) = 1.09, *p* = .58).

#### 3.3 Experimental task.

We employed a validated threat looming paradigm [13] using color photographs of threatening (snakes, spiders) and non-threatening (butterflies, rabbits) animals (40 images per category). Each trial (Fig 2B) began with a fixation cross that disappeared at 300 ms, followed by a looming stimulus that expanded at a constant velocity and disappeared at 1,300 ms. After its disappearance, participants imagined its continued approach and pressed a key at their judged moment of collision. The judged Time-to-Collision (jTTC) was measured from stimulus onset (300 ms) to this key press. A confirmation beep was followed by a jittered inter-trial interval (1,000–3,000 ms). The initial stimulus size (20% or 30% of screen width) and its expansion velocity (determined by one of five actual Time-to-Collision (aTTC) values: 3.0, 3.5, 4.0, 4.5, and 5.0 s) were both varied independently across trials.

The experiment consisted of 160 trials across two blocks, comprising a full factorial design of aTTC (5 levels), stimulus category (4 levels), and initial size (2 levels). Participants completed practice trials using animal silhouettes prior to the main task. The task lasted approximately 35 min, and pupillary responses were recorded throughout.

### 4. Data acquisition and preprocessing

#### 4.1 Behavioral data preprocessing.

Trial-level jTTC data were preprocessed in two stages. First, trials with technical artifacts or incomplete responses were excluded. Second, trials exceeding ±5 SD from each participant’s mean jTTC were classified as outliers and removed. This conservative criterion preserved potential treatment-induced variations while flagging extreme deviations, following methodological recommendations for pharmacological studies [42,43].

#### 4.2 Pupillary data preprocessing.

Pupil size and movement from the right eye were recorded at 2,000 Hz using an EyeLink 1000 Plus system (SR Research) with a display resolution of 1,280 × 1,024 pixels. Participants were positioned 57 cm from the monitor using a chin rest, and a 9-point calibration procedure was performed at the beginning of each experimental block to ensure optimal tracking accuracy.

Pupil size was recorded as pixel area and converted to diameter (*d*) using a validated formula *d* = *αLφ*, where *α* is an empirical scaling factor, *L* denotes the pupil-to-camera distance, and *φ* is the visual angle [44]. Data were preprocessed to remove signal loss, blink artifacts, physiologically implausible values, and statistical outliers beyond ± 5 SD from the trial mean, in accordance with established guidelines [28]. Finally, the cleaned pupillary signals were down-sampled into 10 ms bins to reduce high-frequency noise while preserving physiologically relevant fluctuations [45].

#### 4.3 Stimulus dynamics.

To optimize analysis and integrate looming motion parameters into a single ecologically meaningful measure, we first calculated the approach velocity for each trial using the formula: Velocity = (Screen Width − Initial Width)/aTTC. We then mapped the resulting discrete physical values into a five-level ordinal metric termed “Physical Stimulus Velocity” (PSV). The velocities (179.2–341.3 pixels/s) were categorized into five intervals: very slow (V1: 179.2–211.6), slow (V2: 211.6–244.0), medium (V3: 244.0–276.4), fast (V4: 276.4–308.8), and very fast (V5: 308.8–341.3 pixels/s, right-closed). These PSV levels served as fixed factors in subsequent analyses.

### 5. Analysis framework

#### 5.1 Behavioral analysis.

To quantify the psychophysical characteristics of time perception, we employed a model-based approach: (1) a Linear Model (jTTC = *α* + *β* * aTTC), which served as a baseline assuming a linear relationship between subjective and objective time; (2) a Power Law Model (jTTC = *α* * (aTTC)^*β*), based on Stevens’ Power Law [46], which captures the canonical nonlinear relationship between perception and physical magnitude. In both models, *α* represents scaling parameters, while *β* reflects the rate of change (linear) or degree of nonlinearity (power law) in time perception.

For each participant and condition, model parameters (*α*, *β*) were estimated by iterative optimization, minimizing the sum of squared errors to obtain the best fit. The coefficient of determination (*R*²) was computed for each fit. To evaluate which model best accounted for the data across the dataset, we compared them using the Bayesian and Akaike Information Criteria (BIC and AIC) [47].

Finally, to examine the effects of Treatment, IsThreaten, and Sex on time perception parameters, we constructed Linear Mixed-Effects models using the estimated parameters (*α* and *β*) as dependent variables. Each model included fixed effects of Treatment, Sex, IsThreaten, and their possible two-way interactions. A random intercept for each participant was included to account for individual differences. The significance of the fixed effects was analyzed to determine whether the experimental manipulations modulated the temporal perception parameters.

#### 5.2 Pupil analysis.

**5.2.1. Baseline pupil analysis and correction.** Considering potential pharmacological effects on absolute pupil diameter, we first assessed baseline pupil diameter (0–300 ms) using a two-way ANOVA with Treatment and Sex as factors. All subsequent analyses were baseline-corrected by subtracting the mean baseline value from each time point to isolate task-evoked responses.

**5.2.2. Event-locked analysis.** To examine mean pupillary responses across distinct threat processing phases, ranging from sensory encoding to decision preparation, we defined three temporal windows based on task structure and threat processing dynamics [32,48]:

(1). Stimulus Presentation (300–1,300 ms; encoding of the approaching stimulus during active visual processing); (2). Early Imagination (first 500 ms post-offset; immediate transition to mental representation); (3). Late Imagination (last 500 ms pre-collision; decision-related preparatory processes).

A repeated-measures ANOVA was conducted with Treatment and Sex as between-subject factors, and Phase, IsThreaten, and PSV as within-subject factors. Trials shorter than 2,300 ms (2%) were excluded to ensure complete phase coverage across all windows.

**5.2.3. Time-normalized dynamic analysis.** Direct comparison of pupillary dynamics across trials was precluded by variable trial durations stemming from individual differences in time-to-collision estimation, necessitating the use of temporal normalization to align all trials to a common time scale. Following predefined quality criteria (Supporting information (S1 Text)), 9.65% of trials were excluded, with no differences in the percentage between treatment groups (*χ*²(2) = 0.46, *p* = .53). The remaining trials were processed using piecewise linear time warping to map the fixed stimulus epoch (300–1,300 ms) to [0, 0.5] and the variable imagination epoch to [0.5, 1.0] on a unified scale, followed by smoothing of the normalized time series using cubic B-splines [49–51].

Functional linear mixed models (FLMM) were fitted [52] to examine the influence of Treatment, Sex, IsThreaten, and PSV on pupillary response over normalized time. The initial model followed a full factorial design:


$$\begin{array}{cc} & \text{Baseline}-\text{corrected Pupil Diameter}\sim \text{Treatment}*\text{ Sex}*\text{IsThreaten}*\text{PSV}*\text{Normalized}\_\text{Time}\\ & +(\text{Normalized}\_\text{Time}+\text{IsThreaten}+\text{PSV}|\text{Subject}) \end{array}$$

To ensure interpretability and avoid overparameterization, non-significant higher-order interactions were pruned using likelihood ratio tests [53], resulting in a final model that retained only effects up to three-way interactions.

Together, these analyses provide a comprehensive characterization of pupillary responses: baseline correction isolated pharmacological effects on tonic arousal, event-locked ANOVA identified phase-specific mean differences, and time-normalized FLMM captured full response trajectories across task progress. This multi-faceted approach distinguishes nonspecific drug effects, enables precise stage-specific inference, and reveals holistic temporal dynamics.

#### 5.3 Integrated pattern analysis.

**Fig 3:**
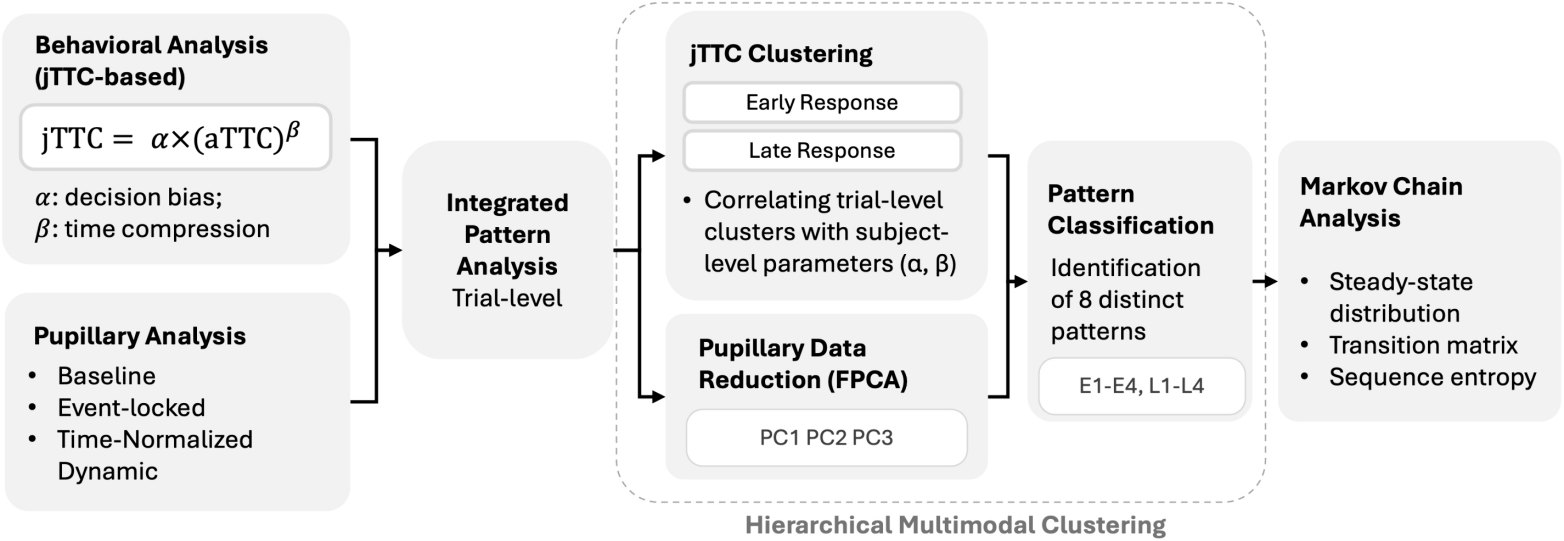
Analysis workflow. **First,** separate behavioral and pupillometric analyses were conducted. Behavioral responses were characterized using the power-law model jTTC = jTTC = *α* * (aTTC)^*β*. Pupillary analysis included baseline pupil size, event-locked responses, and time-normalized dynamics. **Second,** features were integrated through a hierarchical approach: trials were clustered based on jTTC values, with subgroup proportions correlated to subject-level parameters (*α*, *β*). FPCA reduced dimensionality of pupillary data, **followed by** Markov chain analysis to characterize state dynamics, including steady-state distributions, transition matrices, and sequence entropy.

To identify treatment-specific psychophysiological phenotypes that reflect the integrated coordination of behavioral and pupillary systems during threat processing, we developed a comprehensive analytical framework combining FPCA [54], Clustering [55], and Markov chain analysis [56,57] (Fig 3). This approach captures both continuous temporal dynamics and discrete state transitions, aiming to reveal emergent, integrated phenotypes beyond simple correlations between systems. The analysis consisted of three sequential steps:

**5.3.1. Functional Principal Component Analysis (FPCA).** Using FPCA, which better accounts for temporal continuity compared to traditional PCA [58], we extracted principal temporal response patterns (eigenfunctions) from time-normalized pupillary data within each trial. This dimensional reduction approach yielded multiple functional components per trial that collectively characterized the complete temporal dynamics of pupillary responses. We retained principal components that explained over 85% of total variance (individual components >5%) and examined their associations with experimental conditions, individual characteristics, and pupillary features during and after stimulus presentation.

**5.3.2. Hierarchical clustering integrated with jTTC and FPCA components.** To classify trials into distinct response patterns based on their behavioral-physiological characteristics, we integrated the FPCA components with behavioral features (jTTC) using a hierarchical k-means clustering framework. Given their different measurement scales, we first clustered on log-transformed jTTC, then on FPCA components within each behavioral subgroup. Optimal cluster numbers were determined using Calinski–Harabasz index and silhouette coefficients, with clustering stability verified through bootstrap resampling (*n* = 1,000, stability index > 0.85) and cross-validation (accuracy > 0.80). To further determine whether these behavioral subgroups (trial-level) reflected stable computational traits (subject-level), we correlated the individual participants’ proportion of trials in each subgroup with their fitted power law parameters (*α* and *β*).

**5.3.3. Hidden Markov modeling for transition dynamics.** First-order Markov chain analysis were employed to characterize the temporal evolution of response profiles across trials. The analysis focused on three complementary metrics: the steady-state distribution, which reflects the long-term equilibrium probability of each state; the transition probability matrix, which captures the directional dynamics between states; and sequence entropy, which quantifies the complexity and unpredictability of the response sequences. To ensure statistical rigor, Chi-squared tests with standardized residual analysis were utilized to detect specific deviations from expected distributions, while bootstrap resampling (1,000 iterations) was performed to generate 95% confidence intervals for the estimated parameters.

This integrated approach fuses continuous pupillary dynamics with behavioral data, identifying distinct, treatment-modulated response profiles and characterizing their temporal evolution during threat processing.

#### 5.4 Analytic software and correction.

Behavioral analyses and event-locked phase analyses were conducted using SPSS 26. Time-normalized dynamic analyses and integrated pattern analyses were implemented in Python v3.12 using scientific computing libraries (pandas, numpy, scipy, statsmodels, scikit-learn) with custom implementations for functional data analysis. For all analyses, effects were considered significant at *p* < .05 with Greenhouse–Geisser correction for sphericity violations. Multiple comparisons were controlled for false discovery rate using the Benjamini-Hochberg method [59] (*α* = 0.05).

## Results

### 1. Subjective anxiety and potential confounders

**Fig 4:**
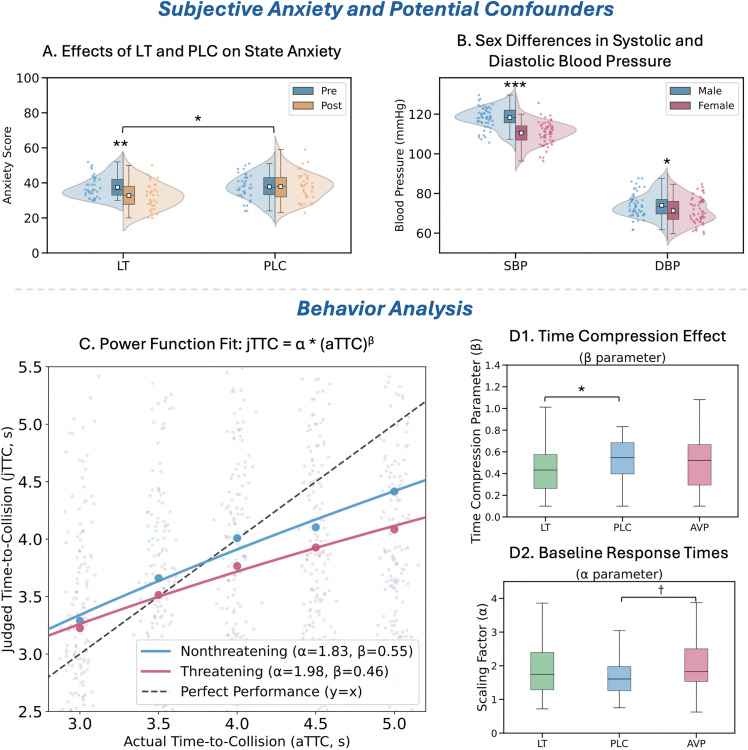
Subjective anxiety, potential confounders, and behavioral results. **(A) State anxiety scores pre- and post-experiment** in the LT and PLC groups. The LT group showed a significant reduction in anxiety at post-test. **(B) Systolic (SBP) and diastolic (DBP) blood pressure measurements**, revealing consistently higher values in males than in females. Both panels are presented as raincloud plots, where points represent mean scores per participant, the central line in the box indicates the median, and the box bounds represent the interquartile range. (C) Nonlinear fitting of aTTC and jTTC using the power-law model jTTC = *α* * (aTTC)^*β*. Participants generally underestimated time-to-collision, with accelerated underestimation as aTTC increased, particularly under threat conditions. Points represent mean jTTC per participant (threat vs. non-threat), jittered (5%) to avoid overlap. The dashed line *y* = *x* indicates accurate estimation. **(D1) Parameter *β*** (temporal scaling: *β* > 1 indicates expansion, *β* < 1 indicates compression). LT group exhibited significantly stronger time compression than PLC. **(D2) Parameter *α*** (general estimation bias: *α* > 1 indicates overestimation, *α* < 1 indicates underestimation). AVP group showed a tendency toward overestimation compared to PLC (*p* = .078). *p < .05,** *p* < .01, ***p < .001, † marginal significance. The data underlying panels A, B, D1, and D2 can be found in S1 Data (Sheet 1 and Sheet 2).

The three groups (LT, PLC, and AVP; *n* = 37 each) did not differ in age, mood, animal fear, or personality traits (all *p*s > .18, S1 Table). A 2 (Treatment: LT versus PLC) × 2 (Sex: male versus female) × 2 (Time: pre versus post) mixed ANOVA with State Anxiety as the dependent variable revealed a significant Treatment × Time interaction (*F* = 4.40, *p* = .039, $\begin{array}{c} \eta_{\text{p}}^{\text{2}}=.\text{0}\text{6} \end{array}$). Between-group comparison showed lower post-task state anxiety level in the LT group compared to PLC (*p* = .017, *d* = 0.51) which was mirrored in within-group analyses indicating significant anxiety reduction in the LT group (pre: 37.51 ± 1.10, post: 33.39 ± 1.36, *p* = .004, *d*z = 0.58) but not in the PLC group (pre: 38.11 ± 1.10, post: 38.09 ± 1.36, *p* = .99, *dz* = 0.02). This may suggest an anxiolytic effect of LT (Fig 4A). No such pattern emerged for AVP (Treatment × Time, *p* = .69). Critically, the anxiety-reducing effect of LT did not differ by sex (*p*s > .18).

Cardiovascular parameters (systolic blood pressure, diastolic blood pressure, and heart rate) were monitored at three time points: baseline, drug peak effect (80 min post-LT; 35 min post-AVP), and post-task. Mixed ANOVAs revealed only a main effect of sex, with males showing higher resting blood pressure than females (SBP: 118.28 ± 0.73 versus 110.30 ± 0.75 mmHg, *p* < .001; DBP: 73.75 ± 0.83 versus 71.15 ± 0.86 mmHg, *p* = .032) (Fig 4B). No significant treatment effects or interactions were found at any time point (all *p*s > .46).

### 2. Time-to-collision analysis

Model comparisons revealed that the power law model provided a superior fit to the linear model in most conditions (69.4%). The compression parameter *β* was consistently below 1 (range: 0.342–0.653, mean = 0.495), together with an average 1.55-fold increase in absolute perceptual error as aTTC increased from 3 to 5s, indicating systematic time compression that accelerated with longer aTTCs. Linear mixed-effects (LME) analyses revealed a significant reduction in *β* (indicating enhanced time compression) for threatening compared to nonthreatening stimuli ((*β* = −0.091, *p* = .06; Fig 4C), under LT compared to PLC (*β* = −0.149, *p* = .033, 95%CI [−0.285–0.012]; Fig 4D1), and in females compared to males (*β* = −0.167, *p* = .016, 95%CI [−0.303–0.032]). No significant reduction in *β* was observed for AVP compared to PLC (*β* = −0.045, *p* = .52).

The scaling parameter *α* (where *α* = 1 indicates accurate estimation) showed substantial individual differences (range: 0.857–2.341, mean = 1.534). LME analyses revealed a trend toward increased *α* (indicating a tendency for time overestimation) under AVP compared to PLC (*β* = 0.504, *p* = .078, 95%CI [−0.057,1.065]; Fig 4D2). No other effects reached significance.

### 3. Pupil analysis

#### 3.1 Baseline analysis.

Analysis of pre-stimulus baseline pupil diameter revealed a significant main effect of treatment (*F* = 4.79, *p* = .01, $\begin{array}{c} \eta_{\text{p}}^{\text{2}}=.\text{0}\text{8} \end{array}$), with the AVP showing larger diameters than the PLC group (*p* = .003), the LT did not significantly differ from either group (*p*s > .073). No sex differences were observed (*p*s > .74).

**Fig 5:**
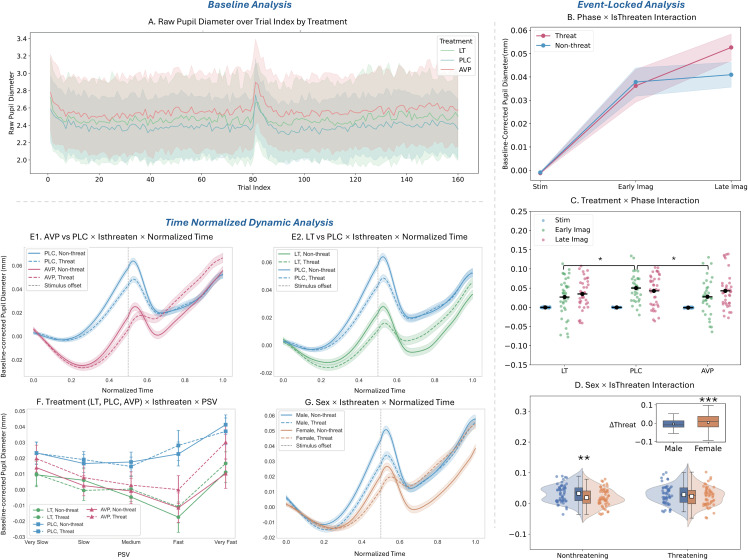
Pupillary measurement: baseline(A), phase-locked (B–D), and normalized time dynamic analyses (E–G). **(A) Baseline pupil size:** The AVP group had a significantly larger uncorrected pupil diameter than the PLC group. The LT group did not differ from either. **(B) Phase × Isthreaten:** Threat evoked a sustained dilation, while non-threat elicited a phasic dilation followed by a plateau. Responses to threat significantly exceeded non-threat in the late imagination phase. **(C) Phase × Treatment:** The PLC group response was significantly larger than both AVP and LT during the early imagination phase. The AVP group exhibited sustained dilation across phases, while PLC and LT responses plateaued from early to late imagination. **(D) Sex × Isthreaten:** Males had significantly larger pupils than females specifically during the non-threat condition (no sex difference under threat). The inset shows a significantly greater threat-specific response (threat–non-threat) in females. **(E1) AVP vs. PLC × Isthreaten × Time:** PLC > AVP during stimulus approach; this pattern reversed to AVP > PLC after stimulus offset under threat. **(E2) LT vs. PLC × Isthreaten × Time:** PLC > LT across time. After offset, threat > non-threat for LT, while PLC showed no threat-non-threat difference. **(F) Treatment × Isthreaten × PSV:** Pupillary velocity showed a V-shaped profile. The nadir occurred earlier for PLC (V3) than for drug groups (V4). AVP showed threat > non-threat from V1 to V5, LT from V3 to V5, while PLC showed no differential sensitivity. **(G) Sex × Isthreaten × Time:** Male > female during stimulus approach; this difference disappeared after stimulus offset under threat. The vertical dashed line at Normalized Time = 0.5 indicates stimulus offset, marking the transition from the perception phase to the imagination phase. Shading/error bars represent SEM. * *p* < .05, ** *p* < .01, ***p < .001. The data underlying panels C and D can be found in S1 Data (Sheet 3 and Sheet 4). The raw data underlying all panels are available in the OSF repository (https://osf.io/hfbn6/).

This pharmacological effect persisted throughout the entire experiment, as confirmed by analysis of uncorrected data across trials (*F* = 3.98, *p* = .023; Fig 5A). After baseline correction, these general differences were eliminated (*p*s > 0.12) allowing examination of task-specific pupillary changes.

#### 3.2 Event-locked analysis.

Analysis of baseline-corrected responses revealed significant main effects of Phase (*F* = 44.33, *p* < .001, $\begin{array}{c} \eta_{\text{p}}^{\text{2}}=.\text{4}\text{6} \end{array}$) and PSV (*F* = 19.88, *p* < .001, $\begin{array}{c} \eta_{\text{p}}^{\text{2}}=.\text{4}\text{4} \end{array}$). Pupils dilated from stimulus presentation to early imagination (Present versus Early: *p* < .001) and stabilized during late imagination (Early versus Late: *p* = .22). Extreme velocities (V1/V5) elicited larger responses than moderate velocities (V2–V4, all *p*s < .01).

A Phase × IsThreaten interaction (*F* = 16.90 *p* < .001, $\begin{array}{c} \eta_{\text{p}}^{\text{2}}=.\text{2}\text{5} \end{array}$; Fig 5B) revealed that threatening stimuli induced progressive dilation across phases (Stim < Early < Late, *p*s < .05), while non-threatening stimuli showed initial dilation followed by plateau (Stim < Early = Late, *p*s < .01). During late imagination, threat-induced pupil dilation exceeded non-threatening responses (*p* = .003).

A Phase × Treatment interaction (*F* = 2.70, *p* = .032, $\begin{array}{c} \eta_{\text{p}}^{\text{2}}=.\text{0}\text{5} \end{array}$; Fig 5C) showed that during early imagination, both LT and AVP reduced responses as compared to PLC (*p*s < .05), while no group differences were observed during stimulus presentation or late imagination (*p*s > .22). Across phases, AVP group showed progressive dilation (Stim < Early Imag < Late Imag, *ps* < .05), LT and PLC groups showed initial dilation followed by plateau (Stim < Early Imag ≈ Late Imag, *p*s < .01).

The IsThreaten × Sex interaction (*F* = 14.20, *p* < .001, $\begin{array}{c} \eta_{\text{p}}^{\text{2}}=.\text{1}\text{2} \end{array}$; Fig 5D) revealed that males showed larger responses than females to non-threatening stimuli (*p* = .005), but no sex differences under threat (*p* = .95). Females exhibited enhanced responses to threatening versus non-threatening stimuli (*p* < .001), while males’ responses remained consistent across both conditions (*p* = .11).

#### 3.3 Time-normalized dynamic analysis.

Extending event-locked findings, functional linear mixed models on baseline-corrected pupil diameter across normalized time revealed more nuanced patterns. Specifically, PSV demonstrated a V-shaped velocity-dependent pattern, with maximal dilation at extreme velocities (V1/V5: *β* = 0.013/0.018, *p*s < .001), moderate dilation at V2 (*β* = 0.005, *p* < .001), and no change at V4 relative to V3 (*p* = .63).

A significant Treatment × IsThreaten × Normalized Time interaction (*β*s = 0.015–0.024, *p*s < .05; Fig 5E1 and 5E2) revealed that during stimulus approach (*t* = 0–0.5), all groups exhibited threat-induced constriction (*p*s < .01), but after stimulus offset (*t* = 0.5–1), threat processing diverged: PLC showed no threat/non-threat difference while both LT and AVP maintained enhanced dilation under threat versus non-threat (*p*s < .001). Notably, between-group comparisons revealed that the LT group maintained overall smaller response than PLC, whereas pupil response was smaller in the AVP compared to the PLC group during stimulus presentation but showed enhanced dilation post-stimulus specifically for threat (*β* = 0.008, *p* < .001).

Furthermore, a Treatment × IsThreaten × PSV interaction (Fig 5F) indicated that the PLC group exhibited the largest overall responses compared to both the AVP and LT groups (*β*s = 0.017–0.035, *p*s < .05). AVP group showed larger responses to threatening versus non-threatening stimuli across all velocities (V1–V5: *β*s = 0.0028–0.0099, *p*s < .001), and LT selectively increased threat responses only at faster velocities (V3–V5: *β* = 0.005–0.0097, *p*s < .001). PLC group showed no velocity-specific threat sensitivity. All groups maintained the V-shaped velocity effect, with drug groups reaching their lowest response at V4 while the PLC group reached its lowest response at V3.

Finally, we found a significant Sex × IsThreaten × Normalized Time interaction (*β* = 0.025, *p* < .001; Fig 5G). During stimulus approach, males showed larger pupil response than females across both threat and non-threat conditions (*p*s < .001). After stimulus offset, however, the sex difference persisted only for non-threatening stimuli (*β* = 0.0221, *p* < .001) and was abolished under threat (*p* = .27). Crucially, females exhibited greater dilation to threatening versus non-threatening stimuli post-stimulus (*β* = −0.0193, *p* < .001), while males showed no threat-related differentiation (*p* = .31).

### 4. Integrated behavioral-pupillary response profiles

#### 4.1. Identification of behavioral-pupillary states.

Eight distinct response profiles were identified through a hierarchical clustering procedure. First, clustering trials based on log jTTC revealed two behavioral subgroups: an early-response group (E: 3.12 ± 0.57 s) and a late-response group (L: 5.80 ± 1.98 s). Correlations with power law parameters confirmed associations with distinct subject-level traits in time estimation: the early-response proportion negatively correlated with both *α* (*r* = −0.45, *p* < .001) and *β* (*r* = −0.36, *p* < .001), indicating systematic underestimation and stronger time compression; conversely, the late-response proportion was positively correlated with both *α* (*r* = 0.45, *p* < .001) and *β* (*r* = 0.36, *p* < .001), indicating the opposite pattern of overestimation and weaker compression.

**Fig 6:**
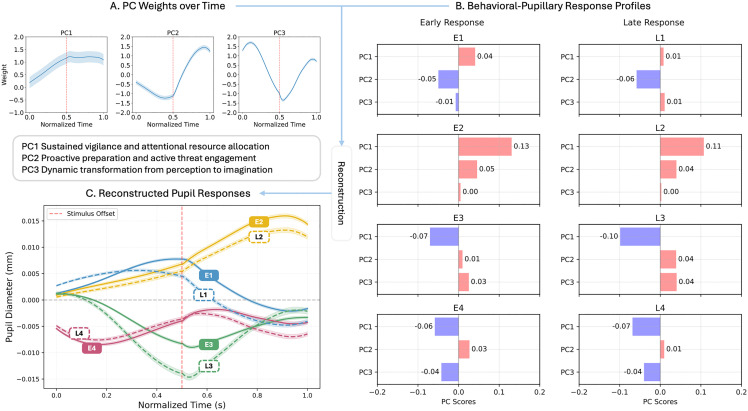
Decomposition and profiling of integrated behavioral-pupillary responses. **(A)** Three dominant functional principal components (PCs) underlying pupillary dynamics. Eigenfunctions *φ*(t) for each component are displayed, with their sign and magnitude defining the functional interpretation: PC1 (Sustained Vigilance) shows sustained positive weighting; PC2 (Proactive Engagement) transitions from negative to positive weighting; PC3 (Cognitive Shift) exhibits a multiphasic (positive-negative-positive) pattern. Individual trial responses are reconstructed as a linear combination of these components: Pupil Diameter(*t*) ≈ Grand Mean(*t*) + (Score₁× *φ₁*(*t*)) + (Score₂ × *φ₂*(*t*)) + (Score₃ × *φ₃*(*t*)). **(B)** Eight distinct response profiles from hierarchical clustering. Profiles were identified by combining behavioral response subgroups (Early: E1–E4; Late: L1–L4) with clustering of PC. **(C)** Reconstructed mean-centered pupillary responses for each profile. To highlight the distinct patterns across conditions, the grand mean trajectory was subtracted. Curves illustrate the characteristic pupillary signature of each profile over time, reconstructed by combining the shared temporal eigenfunctions from (A) with the profile-specific component scores from (B). The error shading represents the 95% confidence interval (CI) estimated via bootstrap resampling (1,000 iterations). The data underlying panel B can be found in S1 Data (Sheet 5).

Second, FPCA decomposed pupillary dynamics into three core components (PC1–3, Fig 6A) accounting for 87.51% of total variance, which were differentially modulated by experimental conditions and individual traits (summarized in Table 1; full analysis in Supplementary Results (S1 Text): Functional Principal Components, S1 and S2 Figs). Based on the full analyses, the components were interpreted as PC1: Sustained Vigilance, PC2: Proactive Engagement, reflecting active preparation for threat, and PC3: Cognitive Shift, capturing the transition from perception to imagination.

**Table 1:** Summarizes the characteristic features and experimental correlations of each component, along with their proposed functional significance.

Principle Component	PC1	PC2	PC3
Variation Explanation	62.67%	17.56%	7.28%
Weight Time Function Features	Early steep rise followed by plateau	Initial inhibition, later strong activation	Early peak, mid-period trough, late recovery
Main Related Pupil Features	Stimulus-Evoked Response (Pupil size during stimulus: *r* = .615, speed of dilating: *r* = .65)	Post-Stimulus Sustenance (Pupil size after stimulus: *r* = .668) vs. Stimulus Suppression (Pupil size during stimulus: *r* = −.35)	Rate Anticorrelation (Slower dilating during stimulus: *r* = −.58, Faster dilating after: *r* = .34)
Experimental Conditions	Female only:	AVP > LT, AVP > PLC	Threat>Nonthreat
	Nonthreatening:	Threat>Nonthreat	V1,V5 < V2-V3
	PLC > LT,PLC > AVP	V1,V2 < V3-V5	
	Threatening: PLC = AVP > LT		
	Velocities: V1,V5 > V2-V4		
	(No male differences)		
Individual Differences	Not significant	Low anxiety > High Anxiety	Early response < Late response
Possible Functional Significance	Sustained Vigilance and Attentional Resource Allocation	Proactive Preparation and Active Threat Engagement	Dynamic Transformation from Perception to Imagination

Integrating behavioral (E/L) and pupillary (PC1–3 scores) features, a second-level clustering yielded eight stable profiles (E1–E4, L1–L4, Fig 6B) that verified by bootstrap (stability index: 0.753 ± 0.125) and cross-validation (0.651 ± 0.114; S3 Fig). These profiles reflect distinguishable threat processing strategies (Table 2).

**Table 2:** Characteristics of the eight behavioral-physiological profiles.

Pattern	Behavioral Association	PC1^a^	PC2^b^	PC3^c^	Interpretation
E1	underestimation tendency (low α); time compression (low β)	**+**	−	−	High vigilance, Disengagement, Perceptual Maintenance
E2		**+**	**+**	~0	High vigilance, Active Engagement, Seamless Transition
E3		−	**+**	**+**	Low vigilance, Active Engagement, Internal Simulation
E4		−	**+**	−	Low vigilance, Active Engagement, Perceptual Maintenance
L1	overestimation tendency (high α); time expansion (high β)	**+**	−	**+**	High vigilance, Disengagement, Internal Simulation
L2		**+**	**+**	~0	High vigilance, Active Engagement, Seamless Transition (similar to E2)
L3		−	**+**	**+**	Low vigilance, Active Engagement, Internal Simulation (similar to E3)
L4		−	**+**	−	Low vigilance, Active Engagement, Perceptual Maintenance (similar to E4)

**Note:** PC scores reflect the polarity of the functional components:

^a^PC1 (Vigilance): (+) denotes High Vigilance (sustained attentional resource allocation); (−) denotes Low Vigilance.

^b^PC2 (Engagement): (+) denotes Active Engagement (proactive preparation for threat); (−) denotes Disengagement.

^c^PC3 (Cognitive Shift): (+) indicates Internal Simulation (shift away from perception); (−) indicates Perceptual Maintenance (focus on sensory input); (~0) indicates Seamless Transition (balanced integration).

For instance, profile E1 was characterized by high vigilance (positive PC1) but disengagement from proactive preparation and a focus on sensory maintenance (negative PC2/PC3), producing a reconstructed pupil response of dilation during approach followed by constriction after stimulus offset. Profile E2 exhibited high vigilance paired with active threat engagement (positive PC1/PC2) and a seamless cognitive transition (neutral PC3), resulting in sustained dilation throughout the task. Notably, late-response profiles L2–L4 mirrored the directional PC patterns of E2–E4 but differed in response magnitude, as shown in Fig 6C.

#### 4.2. Markov chain analysis.

**Fig 7:**
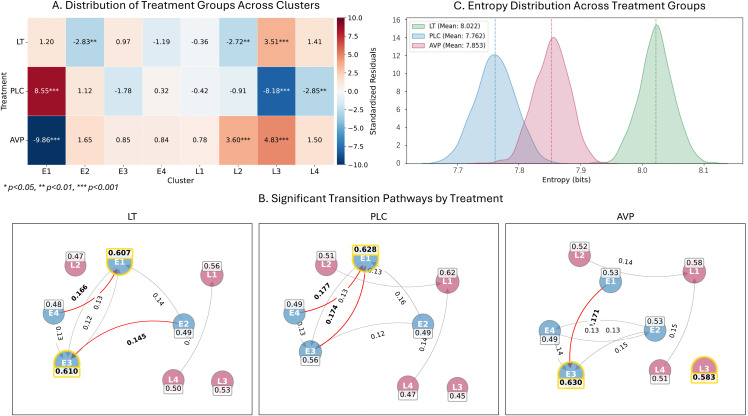
Markov Chain analysis of response patterns. **(A) Stationary distribution revealed treatment-specific deviations from expected state probabilities.** Profile E1 was significantly elevated in PLC and reduced in AVP. Profile L2 was increased in AVP and decreased in LT. Profile L3 was elevated in both LT and AVP but suppressed in PLC. Profile L4 was significantly lower in PLC (* *p* < .05, ** *p* < .01, *** *p* < .001). **(B) Significant within-group transition pathways**. **Nodes** represent distinct hidden states, with boxed numbers indicating self-transition probabilities (persistence). **Yellow halos** identify “attractor states” (high self-transition probability, acting as stability centers). Directed arrows denote transition probabilities between states; **red arrows** highlight significant transitions (*p*-adj < 0.05), while **gray arrows** indicate non-significant pathways (>0.10). **Comparison of topologies:** The PLC group exhibits a convergent structure centered on **E1**, which acts as a dominant attractor receiving significant inflows from other states (e.g., E3 and E4). **AVP treatment alters this dynamic**: the convergent flow into E1 is largely absent (e.g., the E3 → E1 pathway is not significant), and new stability centers emerge at **E3** and **L3** (indicated by yellow halos). The LT group displays an intermediate topology, retaining some inflow to E1 but with reduced connectivity compared to PLC. (See S6 Fig for detailed between-group statistical comparisons of state occupancy and transition probabilities). **Note:** While visual graphs apply a 0.10 visibility threshold, exact between-group differences are detailed in S6 Fig. **(C) Sequence entropy** differed significantly across treatments (LT > AVP > PLC), indicating that PLC produced the most predictable and stable sequences, whereas LT sequences were the most dynamic and unpredictable. **Functional descriptions of key profiles:** Profile **E1** (+, −, −) reflects sustained vigilance paired with inverse proactive engagement and cognitive shift, producing a pupil response of initial expansion followed by contraction. Profile **L2** (+, + , ~0) incorporates vigilance and proactive engagement while bypassing typical cognitive resets, thereby producing uninterrupted pupillary dilation. Profile **L3** (−, + , +) serves as the functional mirror to E1, coupling suppressed tonic arousal with active engagement and switching, characterized by initial contraction followed by recovery.

**4.2.1. Steady state distribution.** Residual analysis revealed a significant main effect of treatment (*χ*²(14) = 333.17, *p* < .001; **Fig 7A**). **Profile E1** (characterized by approach-phase dilation and post-stimulus constriction) was the dominant state in PLC (*z* = 8.75) but suppressed in AVP (*z* = −9.85). **Profile L3** (initial constriction followed by recovery) predominated in the pharmacological conditions, being higher in both AVP *(z* = 4.96) and LT (*z* = 3.53) relative to PLC (*z* = −8.32). **Profile L2** (sustained dilation) further distinguished the two active treatments, being higher in AVP (*z* = 3.57) and lower in LT (*z* = −2.69) **(see** Fig 6 **for each profile’s visual breakdown)**.

The sex effect (*χ*²(7) = 250.84, *p* < .001; S5 Fig) revealed that for late-response profiles (L1–L4), males were higher (*z* = 1.81 to 6.86) while females were lower (*z* = −1.92 to −7.27). Specifically, profile L1 was higher in males (*z* = 6.86) and lower in females (*z* = −7.27), whereas profile E3 showed the reverse (Females: *z* = 7.60; Males: *z* = −7.17). The Isthreaten effect (*χ*²(7) = 65.59, *p* < .001) showed E1 was lower under threat (*z* = −3.17) versus non-threat (*z* = 3.11), while E2 was higher under threat (z = 4.43) than non-threat (*z* = −4.35).

**4.2.2. Dynamics transition.** Analysis revealed significant treatment-specific remodeling of state transition pathways (*χ*² = 699.69, *p*-adj < .001). This remodeling occurred despite consistently high self-transition probabilities (0.45–0.63) across all conditions. Subsequent bootstrap-based comparisons against the PLC baseline showed that both AVP and LT significantly inhibited transitions towards E1 (e.g., AVP: E1–E4 → E1, diff = −0.029 to −0.096, *p*s < .05; LT: E3 → E1, diff = −0.055, *p* = .02) and promote transitions towards L3 (e.g., AVP: L1/L3/L4 → L3, diff = 0.032 to 0.132, *p*s < .05; LT: L1/L4 → L3, diff = 0.042 to 0.045, *p*s < .05). Direct comparison between LT and AVP revealed that AVP significantly promoted transitions towards to L2 (L2 → L2, diff = 0.054, *p* = .04), whereas LT significantly facilitated the transition to E1 (e.g., E4 → E1, diff = 0.058, *p* = .04). Within-group transitions (Fig 7B) further supported these between-group findings and provided a more detailed architecture of significant pathways within each treatment (see S2 and S3 Tables and S6 Fig for full details).

Sex comparisons (*χ*² = 250.85, *p*-adj < .001) revealed males exhibited increased transitions toward L1 (e.g., E1/E3 → L1: *z* = 2.33 to 2.75) while females showed elevated transitions toward E3 (e.g., E1/L4 → E3: *z* = 2.32 to 3.18). A significant threat modulation was also identified (*χ*² = 14.95, *p*-adj = .007), specifically enhancing the E3 → E2 transition under threat (*z* = 2.54).

**4.2.3. Sequence entropy and stability.** Analysis of sequential patterns extended beyond transition probabilities to higher-order Markov entropy (a proxy for sequence flexibility, Fig 7C). The PLC group demonstrated the most rigid dynamics with the lowest entropy (*M* = 7.76 bits, 95% CI [7.70, 7.82]), characterized by repetitive, E1-centric sequences (e.g., E1 → E1 → E1, *z* = 5.13, *p* < .001). In contrast, pharmacological treatments significantly increased entropy, reflecting enhanced system flexibility though via distinct magnitudes. The LT group exhibited the highest entropy (M = 8.02 bits, 95% CI [7.96, 8.08]), significantly exceeding both AVP and PLC (*p*s < .001), indicating a generalized state of maximal unpredictability. The AVP group showed intermediate entropy (*M* = 7.85 bits, 95% CI [7.80, 7.91]), significantly differing from both PLC and LT (*p*s < .001), consistent with its specific shift toward Profiles E3 and L3 rather than global randomness.

Additionally, females showed lower entropy than males (diff = −0.05, *t* = 42.55, *p* < .001), and threat induced a subtle reduction in entropy compared to non-threat (diff = −0.02, *t* = 20.13, *p* < .001), sugges*t*ing increased sequence regularity.

## Discussion

Using a randomized, placebo-controlled design that integrated behavioral and eye-tracking measures, we investigated the processing of looming threats during both sensory approach and internal imagination, revealing that vasopressin (AVP) and angiotensin II (blocked via LT) modulate the distinct temporal stages of threat evaluation by regulating sustained vigilance and adaptive flexibility.

Our findings reveal three key aspects:

1. Threat-induced time compression: while confirming that looming threats lead to underestimated collision times [13], our computational modeling revealed this effect is driven by a non-linear compression of temporal sensitivity (*β*), rather than a shift in decision bias (*α*), with further modulation by sex.
2. Dynamic pupillary responses during approaching threat and occlusion: compared to non-threat, threat stimuli elicited a biphasic pupil pattern of initial sustained constriction during approach (minimum at disappearance) followed by progressive dilation prior to the expected (imagined) collision. This pattern suggests a dynamic shift from vigilance to defensive preparation.
3. AVP and LT produced dissociable psychophysiological profiles: AVP promoted a more rigid, hyper-vigilant strategy, characterized by a subjective temporal expansion (*α* > PLC), heightened pupillary responses to imagined threats, and an arousal system locked into high-cost, sustained states (L2). In contrast, LT fostered a more flexible, energy-conserving strategy, manifested as heightened time compression (*β* < PLC) and globally attenuated yet threat-discriminative pupillary responses. Crucially, LT exhibited the highest cognitive flexibility (indexed by peak sequence entropy) and effectively suppressed transitions toward the metabolically costly L2 state.

### 1. Threat-induced time compression of looming threat

Building on findings that threat induces an underestimation of collision time [13], our modeling reveals this effect is driven by temporal compression (*β*) rather than subjective scaling (*α*). Specifically, threat influences time perception by selectively compressing longer intervals [60,61]. This creates a subjective sense of immediacy, ensuring distant threats are perceived as imminent to facilitate preemptive defense [62,63].

Pupillary responses revealed a distinct biphasic pattern corresponding to the task phases. The visual approach was characterized by sustained constriction until stimulus offset. This progressive constriction likely reflects active content decoding and a cognitively driven near reflex for foveation, signaling an integrated engagement of cognitive and oculomotor systems [64,65]. Following stimulus offset, however, a distinct transition to progressive dilation occurred, suggesting a shift from external sensory processing to internal simulation. This phase involves increasing autonomic arousal and anticipatory defense as the imagined collision nears [48,66], while the stimulus trajectory is actively maintained in working memory.

Approach velocity modulated these responses in a V-shaped pattern. Maximum constriction occurred at intermediate velocities (V2 for threat, V4 for non-threat) while the slowest (V1) and fastest (V5) velocities elicited greater dilation. We hypothesize this pattern reflects a shift in defensive strategies based on spatial imminence [67] in which detailed sensory analysis is prioritized at intermediate speeds via constriction [28], whereas extreme velocities drive arousal shifts under conditions of either low or high perceived relevance [68,69]. These findings underscore a continuous interaction between looming and semantic content, aligning with broader models of threat processing along the imminence spectrum [34,67].

Pattern analysis revealed a clear functional divergence: non-threat engaged Profile E1 whereas threat recruited Profile E2, a distinction evidenced by their steady-state distributions, transition matrices, and sequential entropy levels (Fig 7A–7C). Interpreting these profiles via principal component loadings and reconstructed pupillary dynamics (Fig 6A–6C; Table 2) uncovers their distinct operational modes. Specifically, Profile E1 (+, −, −) functions as a low-cost surveillance mode, characterized by sustained vigilance (PC1+) yet reduced proactive threat engagement (PC2−) and an absence of cognitive shift towards internal simulation (PC3−). This pattern is physiologically reflected in an initial pupillary expansion followed by rapid contraction. In contrast, Profile E2 (+, + , ~0) indicates a high-alert mode that incorporated vigilance and proactive threat engagement while bypassing the typical cognitive reset (neutral PC3). Consequently, E2 likely facilitates a nearly seamless transition from perception to imagination that drives persistent, uninterrupted pupillary dilation throughout the task.

This separation reflects a likely balance between metabolic efficiency and survival efficacy [70]. The preference for E1 under non-threat conditions suggests a safety-driven downregulation of cognitive load to support adaptive resource conservation [71], whereas the recruitment of E2 may prioritizes survival in the face of potential danger [72]. This interpretation is furthermore reflected in the *α* and *β* parameters: both profiles correlate negatively with these parameters, confirming that the “early” nature of E1 and E2 translates into a behavioral underestimation of time-to-collision. This tendency, while present in the efficiency-oriented E1, is significantly amplified by the survival-optimized processing of E2, bridging our trial- and group-level findings.

### 2. Sex differences in threat processing strategies

Sex modulated the threat processing architecture, with females exhibiting heightened temporal compression (*β*: female < male). This suggests a biologically ingrained precautionary perceptual bias rather than a conscious strategy, favoring an overestimated imminence of collision to ensure accelerated defensive responses [73,74].

Pupillometric analyses further differentiated these strategies. Males exhibited larger pupil diameters than females specifically during non-threatening contexts and lacked valence-based differentiation, suggesting a tonic state of generalized vigilance [29]. In contrast, females showed a significant threat-specific upregulation (threat > non-threat) to reach arousal levels comparable to males particularly under imaginary threat. This female-specific dynamic likely reflects the enhanced engagement of internal simulation processes [75,76].

Pattern analysis further substantiates this physiological dissociation (see Supplementary Discussion (S1 Text) and Fig 6A–6C). Specifically, males showed a bias toward Profile L1 (+, −, +), a strategy broad monitoring that prioritizes generalized environmental readiness at the cost of detailed evaluation. Conversely, females preferentially engaged Profile E3 (−, + , +), a strategy characterized by suppressed generalized vigilance in favor of sustained internal modeling and proactive engagement. This phasic resource investment facilitates the superior threat discrimination and early precautionary responses observed behaviorally [77,78].

### 3. Neuropeptide modulation of the looming response

#### 3.1 The placebo baseline: A resource-conserving mode for fading threat cues.

Under placebo, pupillary threat discrimination (threat < non-threat) was evident during stimulus presentation but dissipated in the subsequent imagination phase (Fig 5E1–5E2). Unpacking this dynamic through Markov chain reveals that the PLC group was anchored in Profile E1 (+, −, −). This dominance is robustly evidenced by a high steady-state probability, preferential transition bias, and the lowest sequential entropy (Fig 7A–7C), indicating a stable and predictable mode. Characterized by sustained vigilance (PC1+) but suppressed proactive engagement (PC2−) and internal simulation (PC3−), this E1 configuration produced a reconstructed pupil response marked by an initial expansion followed by a pronounced contraction (Fig 6A–6C).

We interpret the PLC group’s dominance in Profile E1 as reflecting a default neurophysiological tendency to conserve effort rather than endogenously sustaining vivid internal representations without sensory support. Consistent with the dual modes of the LC-NE system [71,79], the E1 trajectory suggests that the preserved phasic response (expansion) may reflect adequate reactivity to the immediate external cue, while the post-stimulus rapid decay reflects a deficiency in the sustained tonic LC-NE activity required to maintain internal cognitive representations. Thus, in the placebo condition, the brain appears to adopt a resource-conserving strategy: it responds competently to the present threat but defaults to a lower-energy state once external support is withdrawn, allowing the internal threat representation to fade and physiological arousal to gradually return to baseline.

#### 3.2 Vasopressin promotes a hyper-driven, enhanced simulation mode.

Behaviorally, AVP administration increased the subjective scaling (*α*), reflecting a perceptual expansion of time. This effect is likely driven by AVP-induced physiological arousal which accelerates the internal clock’s pacemaker [80], causing a higher accumulation of temporal units that leads to overestimated collision times and delayed motor responses.

Pupillometry revealed a tripartite signature: AVP elevated baseline (tonic) arousal, attenuated the phasic dilation to the approaching threat stimulus (likely reflecting a ceiling effect consistent with the Law of Initial Value or active sensory gating [81,82]), and most critically, sustained elevated dilation during the occlusion phase under threat. This pattern implies a strategic trade-off: resources are diverted from immediate, bottom-up stimulus processing to the top-down maintenance of the internal threat model after the external cue fades, reflecting a sustained allocation of cognitive effort [75,83].

Markov chain analysis further underscored this resource reallocation. Relative to PLC, AVP altered the system’s engagement by significantly inhibiting the PLC-dominant Profile E1 (+, −, −), a low-effort state marked by rapid decay of the internal representation. Instead, AVP primarily promoted transitions toward Profile L3 (−, + , +), which serves as the functional opposite to E1. By coupling low tonic arousal with high preparatory focus and active switching, L3 reflects a resource-efficient strategy of dynamic simulation that ensures the threat representation is actively updated rather than allowed to decay. This aligns with the reconstructed pupillary trajectory of initial contraction followed by recovery. Moreover, compared to LT, AVP significantly promoted transitions toward Profile L2 (+, + , ~0). Physiologically, L2 mirrors the threat-driven Profile E2—sharing its high tonic vigilance and proactive preparation that facilitate a seamless perception-to-imagery transition, evidenced by continuous pupil dilation. However, unlike the premature reaction in E2, L2 is associated with a significantly longer time-to-collision estimation (Late Response). This dissociation implies that AVP functions by temporally extending the window of high vigilance: rather than triggering an immediate release, AVP sustains the intense physiological readiness of E2 throughout the delay. Consequently, this dual mechanism of dynamic simulation via L3 supported by the sustained vigilance of L2 allows AVP to maintain the threat model through both active updating and stable guarding. This suggests that the behavioral “waiting” observed under AVP is not a passive delay, but a resource-intensive maintenance of the unseen threat. By shielding the internal representation from decay, AVP effectively optimizes the cognitive state for a conservative, survival-oriented decision policy.

Finally, although the peripheral administration of a peptide like AVP—with its debated blood-brain barrier permeability—raises questions about central bioavailability, the significant behavioral and pupillary effects observed here align with a growing body of evidence indicating that peripherally administered neuropeptides can exert central actions through direct as well as indirect mechanisms, such as vagal nerve activation or engagement of receptor-mediated transport systems (details see also [84–86]).

#### 3.3 Losartan induces a more controlled processing mode.

LT reduced subjective state anxiety in the absence of changes in peripheral cardiovascular activity. This pattern may reflect a delayed peak efficacy of LT on cardiovascular function in normotensive subjects [20,21] and the differentiation of systems underlying subjective experience and autonomic reactivity or threats [2,87]. While the central blood-brain barrier penetration of LT remains debated our findings are in line with previously reported effects in human psychopharmacological studies and models suggesting direct effects via blood-brain barrier crossing and/or via actions on circumventricular organs and subsequent modulation of central autonomic and stress circuits, or peripheral effects mediating the anxiolytic responses [20,21,88–90].

Behaviorally, this reduction in anxiety translated into a distinct computational strategy: whereas AVP modulated subjective scaling (*α*) to enforce a wait-and-see bias, LT acted to sharpen the temporal compression (*β* < PLC). Intriguingly, although this enhanced compression mirrors the directional shift observed under threat conditions and in female participants, it likely stems from a fundamentally different driver—representing a restorative optimization rather than the reactive mobilization typical of threat-induced overestimation [62,63].

The physiological basis was further supported by pupillometry effects, which revealed that LT reduced overall autonomic arousal (smaller pupil responses) while preserving post-stimulus threat discrimination, particularly at high approach velocities. This specific preservation may imply a precision-adaptation process [70], reflecting that LT optimized the signal-to-noise ratio.

Markov chain analysis further clarified how LT optimized this architecture. Like AVP, LT shifted the system away from the PLC-dominant E1 towards the dynamic simulation strategy of Profile L3, but it did so by actively suppressing (rather than anchoring) the high-tonic vigilance state L2. This disengagement biased the system towards a state of highest sequence entropy and transition diversity reflecting a state of cognitive flexibility or metastability [91,92].

Given that anxiety is often characterized by cognitive rigidity [93], where neural circuits become locked in maladaptive, high-arousal loops, LT appears to counteract this by promoting a state of flexible switching between internal states, recruiting L3 only when needed and otherwise relaxing into lower-energy configurations. This may reflect that LT promotes a state of high flexibility and low metabolic cost, that prevents hyper-arousal while keeping neural dynamics fluid to cope with threats.

Despite these distinct computational trajectories, both neuropeptides converged on a common physiological effect: a selectively heightened pupil response to threat versus non-threat stimuli during the post-stimulus imaginary phase, absent under placebo. Integrating these behavioral-physiological findings, suggests distinct therapeutic potential with AVP acting as “Active Shield” that maintains protection via structural rigidity and high energy expenditure, and LT as an “Adaptive Optimizer,” that reduces the physiological costs of anxiety to enable le a streamlined, flexible path to action.

### 4. Limitations

Our study has several limitations. First, while we analyzed pupillary and behavioral responses, we did not examine their link to neural activation patterns. Future work could combine pupillometry with neuroimaging to directly map these relationships. Second, although chromatic differences existed between stimulus phases (colored versus grayscale), our key findings—such as threat-specific pupillary dilation and pharmacological modulation—were consistent across matched conditions, suggesting cognitive-emotional rather than light reflex. Nevertheless, strictly controlled visual properties in future designs would strengthen validity. Third, to achieve model parsimony, we operationalized looming using the composite variable PSV treated categorically. While effective for capturing robust trends, this discretization precludes characterizing the fine-grained, continuous psychophysical function relating approach speed to physiological responses. Furthermore, the composite nature of our metric limits the ability to dissociate which specific sensory feature (e.g., angular velocity, rate of retinal expansion, or direct time-to-collision estimation) predominantly drives the observed effects. Future research employing continuous and orthogonal manipulation of these optical parameters is needed to pinpoint the primary neural encoding principle for looming threat in humans, akin to findings in other species [94]. Finally, our principal component interpretations, while aligned with theoretical frameworks (e.g., arousal modulation, threat detection networks), remain inferential. Empirical validation of these components’ neural substrates is needed.

## Supporting information

S1 Fig Principal component analysis of pupillary features. **(A)** Temporal weight functions showing the contribution of PC1–3 over normalized time. The solid lines represent the mean weights, with shaded areas indicating the 95% confidence intervals. The vertical dashed red line marks the stimulus onset. **(B)** Scree plot displaying both individual (blue bars) and cumulative (red line) explained variance for the first three PCs. PC1, PC2, and PC3 account for 62.7%, 17.6%, and 7.3% of the total variance respectively, with a cumulative explained variance of 87.6%. **(C)** Reconstructed pupil diameter changes based on PC weights × PC scores for PC1–3. For each PC, the solid line represents pupil diameter changes at +2 SD of PC scores, the dotted line at −2 SD, and the dashed line shows the mean response. Changes in pupil diameter are shown in millimeters (mm). The data underlying panel B can be found in S1 Data (Sheet 6).
(TIF)

S2 Fig Relationships between pupillary principal components, experimental conditions, and individual differences. **(A)** Effects of sex, treatment, and threat conditions on PC1 scores: **(A1)** No significant differences among treatments in males; **(A2)** In females, PLC group showed higher PC1 scores than drug groups under non-threatening conditions, while PLC and AVP groups showed higher scores than LT group under threatening conditions. **(B)** PC2 scores across conditions: AVP group significantly higher than PLC and LT groups, and threatening stimuli higher than non-threatening stimuli; AVP and LT groups showed increased PC2 scores under threat, while PLC group remained unchanged. **(C)** Principal component scores across different speeds: PC1 sensitive to extreme speeds (V1,V5), PC2 increased with speed, PC3 more sensitive to medium speeds (V2–V4). **(D)** Negative correlation between PC2 and state anxiety change (Post-Pre) (*r* = −0.302, *p* = .001). **(E)** Positive correlation between PC3 and judged time-to-collision (jTTC) (*r* = 0.263, *p* < .01). **(F)** Correlations between principal components and pupillary parameters: PC1 positively correlated with pupil dilation during stimulus presentation, PC2 positively correlated with post-stimulus pupillary changes but negatively with during-stimulus pupil state, PC3 negatively correlated with during-stimulus change rate (*r* = −0.580) and positively with post-stimulus change rate (*r* = 0.337). (*p < .05, **p < .01, ***p < .001). The data underlying panels A1, A2, B, and C can be found in S1 Data (Sheet 7).
(TIF)

S3 Fig Classification and validation of temporal-dynamic patterns in pupillary response. Top Left: Distribution of log-transformed response time (Log-jTTC), showing two natural groupings of early response (blue) and late response (orange). Top Right: Clustering validation metrics (Silhouette Score and Normalized CH Score) across different numbers of clusters (2–5), showing increasing CH Score with higher cluster numbers. Bottom Left: Bootstrap stability index distribution (red dashed line: mean of 0.753; green dashed lines: ±1 standard deviation). Bottom Right: Five-fold cross-validation stability scores (red dashed line: mean stability of approximately 0.7).
(TIF)

S4 Fig Principal component analysis of identified response patterns. Left: Distribution of data points in PC1-PC2 space with color-coded clusters representing different response patterns. Right: Heatmap displaying PC1, PC2, and PC3 values for each of the eight identified patterns (E1–E4: early response patterns; L1–L4: late response patterns), showing principal component characteristics for each cluster.
(TIF)

S5 Fig Standardized residuals of factors across response pattern clusters. Chi-squared and residual analysis examining differential distributions across early (E1–E4) and late (L1–L4) response patterns for: Treatment (top left: LT, PLC, AVP); Sex (top right: Male, Female); IsThreaten (bottom left: Nonthreatening, Threatening); and Physical Stimulus Velocity (PSV) (bottom right: V1–V5). Values represent standardized residuals, with |*z*| > 1.96 indicating significantly higher or lower frequencies than expected by chance. Cramer’s *V* values indicate effect size for each factor.
(TIF)

S6 Fig Treatment group differences in transition probabilities. Each matrix displays pairwise comparisons of transition probabilities between groups: PLC – LT (left), PLC – AVP (middle), and AVP – LT (right). Rows represent the current state (*t*) and columns represent the next state (*t* + 1). Cell colors indicate the magnitude and direction of the probability differences, with red indicating a higher probability in the first group (positive difference) and blue indicating a higher probability in the second group (negative difference). Black boxes highlight transitions exhibiting robust differences based on bootstrap analysis (95% confidence intervals of the difference excluding zero).
(TIF)

S7 Fig Residual analysis: original vs. reconstructed pupil responses. The solid blue line represents the grand mean of the raw data (with the blue shaded region indicating the standard error of the mean), and the dashed purple line shows the grand mean reconstructed by the FPCA model. The near-perfect overlap between these two trajectories confirms that the model is unbiased. The solid gray line represents the mean of the residuals, which remains flat at zero. The surrounding gray shaded region indicates the spread of the residuals, reflecting the high-amplitude spontaneous physiological unrest (e.g., hippus) and measurement noise characteristic of pupillometry. The FPCA model functions as a data-driven filter, effectively isolating the task-locked cognitive signal from this background noise.
(TIF)

S1 Table Demographics and questionnaire scores between treatment groups. (PDF)

S2 Table Within-treatment transition probabilities and 95% confidence intervals from bootstrap analysis (1,000 iterations). (PDF)

S3 Table Between-treatment differences in transition probabilities and 95% confidence intervals from bootstrap analysis (1,000 iterations). (PDF)

S1 Text Supplementary methods and analyses. Details regarding drug administration, randomization procedures, intermediate results, and additional statistical analyses. A supplementary discussion on sex differences is also included.
(DOCX)

S1 Data Underlying numerical data. Excel spreadsheet containing the individual subject-level data used to generate Figs 4A, 4B, 4D1, 4D2, 5C, 5D, 6B, S1B, S2A1, S2A2, S2B, and S2C. Specific sheets are referenced in the corresponding figure legends. The complete dataset is available at https://osf.io/hfbn6/.
(XLSX)

S1 File Trial protocol. (PDF)

S2 File Institutional Review Board (IRB) documentation. (PDF)

S1 Checklist CONSORT 2025 checklist (Hopewell S, and colleagues. BMJ. 2025; 388:e081123, https://dx.doi.org/10.1136/bmj-2024-081123). This checklist is licensed under the Creative Commons Attribution 4.0 International License (CC BY 4.0; https://creativecommons.org/licenses/by/4.0/).
(PDF)

## Abbreviations

AIC: Akaike Information Criterion
aTTC: Actual Time-to-Collision
AVP: Arginine vasopressin
BIC: Bayesian Information Criterion
FLMM: functional linear mixed models
FPCA: Functional Principal Component Analysis
jTTC: Judged Time-to-Collision
LC-NE: locus coeruleus-norepinephrine
LME: linear mixed-effects
PANAS: Positive and Negative Affect Schedule
PLC: Placebo
PSV: Physical Stimulus Velocity
RAS: Renin-Angiotensin System
