# Supplementary material for: Vasopressin and angiotensin II pathways differentially modulate human fear response dynamics to looming threats
Source: PLoS Biol. 2026 Feb 24;24(2):e3003668. doi: 10.1371/journal.pbio.3003668 (PMC12978571; doi:10.1371/journal.pbio.3003668)
Supplement: S1 Table — (PDF) [file pbio.3003668.s008.pdf]

S1 Table. Demographics and questionnaire scores between treatment groups.

Variables	LT	PLC	AVP	*p*-value
Age, Years	21.89 ± 2.31	22.19 ± 2.54	21.62 ± 2.10	0.58
PANAS_positive	29.49 ± 4.77	27.54 ± 5.29	27.51 ± 6.40	0.22
PANAS_negative	15.49 ± 5.18	16.19 ± 4.92	14.54 ± 5.72	0.41
TAI	37.86 ± 7.63	40.22 ± 6.80	37.11 ± 8.17	0.19
SAI-Baseline	37.51 ± 6.08	38.14 ± 7.24	35.30 ± 7.31	0.18
SAI-Post	33.41 ± 8.26	38.11 ± 8.02	34.49 ± 9.34	0.05
LSAS_Anxiety	47.57 ± 12.22	45.62 ± 8.57	43.27 ± 9.14	0.19
LSAS_avoidance	44.19 ± 11.90	43.84 ± 10.29	41.22 ± 8.33	0.40
AFQ-Rabbit	6.32 ± 1.31	6.54 ± 1.30	6.41 ± 1.91	0.83
AFQ-Butterfly	7.89 ± 3.56	8.46 ± 3.32	7.78 ± 2.75	0.63
AFQ-Snake	18.89 ± 4.69	17.92 ± 5.27	18.03 ± 5.01	0.66
AFQ-Spider	19.62 ± 5.09	20.08 ± 4.54	18.97 ± 5.37	0.63

Note: Values are presented as mean ± SD. The *df* for *F* values is 108.

PANAS, Positive and Negative Affect Schedule; LSAS, Liebowitz Social Anxiety Scale; TAI and SAI, Trait and State subscales of the State-Trait Anxiety Inventory; AFQ, Animal Fear Questionnaire; LT, losartan; PLC, placebo; AVP: vasopressin.
