# Supplementary material for: Vasopressin and angiotensin II pathways differentially modulate human fear response dynamics to looming threats
Source: PLoS Biol. 2026 Feb 24;24(2):e3003668. doi: 10.1371/journal.pbio.3003668 (PMC12978571; doi:10.1371/journal.pbio.3003668)
Supplement: S2 Table — (PDF) [file pbio.3003668.s009.pdf]

S2 Table. Within-treatment estimated transition probabilities and 95% confidence intervals from the bootstrap analysis (1000 iterations).

Treatment	Transition	Probability	[95% CI]	Sig.
LT	E3 → E3	0.610	[0.584, 0.632]	*
	E1 → E1	0.607	[0.582, 0.630]	*
	L1 → L1	0.563	[0.531, 0.595]	*
	L3 → L3	0.529	[0.488, 0.568]	*
	L4 → L4	0.497	[0.458, 0.533]	*
	E2 → E2	0.493	[0.455, 0.529]	*
	E4 → E4	0.484	[0.447, 0.516]	*
	L2 → L2	0.473	[0.430, 0.512]	*
	E4 → E1	0.166	[0.141, 0.190]	*
	E2 → E3	0.145	[0.121, 0.172]	*
PLC	E1 → E1	0.628	[0.607, 0.649]	*
	L1 → L1	0.616	[0.584, 0.646]	*
	E3 → E3	0.557	[0.529, 0.582]	*
	L2 → L2	0.511	[0.471, 0.551]	*
	E2 → E2	0.494	[0.462, 0.527]	*
	E4 → E4	0.491	[0.457, 0.524]	*
	L4 → L4	0.467	[0.428, 0.506]	*
	L3 → L3	0.450	[0.385, 0.512]	*
	E4 → E1	0.178	[0.154, 0.202]	*
	E3 → E1	0.175	[0.156, 0.195]	*
AVP	E3 → E3	0.63	[0.606, 0.653]	*
	L1 → L1	0.584	[0.553, 0.614]	*
	L3 → L3	0.583	[0.545, 0.619]	*
	E1 → E1	0.532	[0.498, 0.564]	*
	E2 → E2	0.528	[0.493, 0.559]	*
	L2 → L2	0.522	[0.488, 0.558]	*
	L4 → L4	0.509	[0.474, 0.545]	*
	E4 → E4	0.493	[0.458, 0.528]	*
	E1 → E3	0.172	[0.150, 0.195]	*
	L4 → L1	0.152	[0.129, 0.177]	*

Note: * indicates a significant difference where the 95% bootstrap confidence interval does not contain zero
