# Supplementary material for: Vasopressin and angiotensin II pathways differentially modulate human fear response dynamics to looming threats
Source: PLoS Biol. 2026 Feb 24;24(2):e3003668. doi: 10.1371/journal.pbio.3003668 (PMC12978571; doi:10.1371/journal.pbio.3003668)
Supplement: S3 Table — (PDF) [file pbio.3003668.s010.pdf]

S3 Table. Between-treatment differences in transition probabilities and 95% confidence intervals from bootstrap analysis (1000 iterations)

Treatment	Transition	Diff.	[95% CI]	Sig.
PLC - LT	E3 → E1	0.054	[0.033, 0.079]	*
	L1 → L3	-0.045	[-0.064, -0.025]	*
	L4 → L3	-0.042	[-0.069, -0.016]	*
PLC - AVP	L3 → L3	-0.132	[-0.201, -0.063]	*
	E1 → E1	0.096	[0.058, 0.135]	*
	E3 → E1	0.075	[0.053, 0.099]	*
	E3 → E3	-0.073	[-0.107, -0.040]	*
	E4 → E1	0.070	[0.037, 0.101]	*
	E2 → E1	0.060	[0.029, 0.089]	*
	L3 → E3	0.053	[0.017, 0.090]	*
	L1 → L3	-0.051	[-0.069, -0.033]	*
	E4 → E2	-0.039	[-0.066, -0.012]	*
	E1 → E3	-0.039	[-0.066, -0.012]	*
	E2 → E4	-0.039	[-0.067, -0.011]	*
	L3 → E1	0.039	[0.009, 0.068]	*
	L3 → E2	0.034	[0.007, 0.063]	*
	L4 → L3	-0.032	[-0.057, -0.007]	*
	L4 → E1	0.029	[0.006, 0.052]	*
AVP - LT	E1 → E1	-0.074	[-0.115, -0.033]	*
	E4 → E1	-0.058	[-0.089, -0.029]	*
	E1 → E3	0.043	[0.015, 0.070]	*
	L3 → E3	-0.041	[-0.067, -0.015]	*
	E4 → E2	0.040	[0.011, 0.068]	*
	E4 → L2	0.022	[0.005, 0.041]	*
	L2 → L2	0.054	[0.032, 0.078]	*

Note: * indicates a significant difference where the 95% bootstrap confidence interval does not contain zero
