# Supplementary material for: Vasopressin and angiotensin II pathways differentially modulate human fear response dynamics to looming threats
Source: PLoS Biol. 2026 Feb 24;24(2):e3003668. doi: 10.1371/journal.pbio.3003668 (PMC12978571; doi:10.1371/journal.pbio.3003668)
Supplement: S1 Text — Details regarding drug administration, randomization procedures, intermediate results, and additional statistical analyses. A supplementary discussion on sex differences is also included. (DOCX) [file pbio.3003668.s011.docx]

**Method**

**Participant**

**Participants were excluded if they met any of the following conditions:**

- History of hepatic, renal, gastrointestinal, respiratory, endocrine, neuropsychiatric, cardiovascular diseases.
- A history of cardiac disease including arrhythmias; history of syncope or unexplained loss of consciousness; history of renal stones or renal failure; history of diabetes mellitus or diabetes insipidus.
- History of or present renal artery stenosis (reduced blood flow to the kidneys).
- History of hypersensitivity or allergic reaction to any medication or hormone, strong allergic reaction to food, or general tendency for allergies.
- Known hypersensitivity to losartan or related agents (e.g. telmisartan), antidiuretic hormone or any other component of the formulation; known hypersensitivity to any other angiotensin II receptor antagonist.
- Subjects with hypertension (BP > 140/90mmHg) or hypothension (BP < 90/60mmHg).
- Current hyponatremia (low levels of sodium in the blood) (symptoms include nausea, fatigue and weakness or swelling of hands, feet or legs)
- History of alcohol or drug abuse；smoker (≥ 10 cigarettes or ≥ 3 cigars or ≥ 3 pipes/day)
- Blood donation (≤ 1 month prior to administration)
- Take oral contraceptives or receive hormonal medications in the three months prior to the experiment.
- Pregnant or breastfeeding.
- Current disorder, including infections such as COVID, flu or cold.

**Participant Pre-screening Procedures**

Prior to participation, all volunteers underwent an electrocardiogram (ECG) examination to rule out potential cardiovascular contraindications. To minimize potential confounds from psychoactive substances, participants were required to abstain from caffeine and alcohol for 24 hours prior to the testing session.

For female participants, a urine-based pregnancy test was administered on the day of the experiment to exclude pregnancy. Additionally, the phase of their menstrual cycle was documented based on self-report following established procedures [1,2] to account for potential hormonal influences.

Based on these pre-screening procedures, which applied stricter, study-specific health criteria, 82 volunteers were excluded from participation. Among them, 62 were excluded due to abnormal or potentially abnormal ECG findings, 17 due to blood pressure outside the target range (systolic >130 or diastolic <80 mmHg), and 3 due to abnormal resting heart rate (<60 or >90 bpm).

**Drug**

**Drug Preparation and Administration Protocol**

Argipressin (AVP; Bio-Techne China Co., Ltd) was aliquoted and stored at -20°C until the day of the experiment. For administration, AVP was dissolved in a sterile vehicle solution of saline and glycerol, passed through a 0.22 μm Millipore filter to ensure sterility, and loaded into spray bottles. Placebo sprays were prepared in an identical manner but omitted the active AVP peptide.

Following our validated protocol [3,4], each participant received six separate 0.1 ml puffs (total volume = 0.6 ml), with a 30-second retention period between puffs. The puffs were administered in an alternating fashion: three puffs were directed to the superior surface of the tongue and three to the inferior surface. Participants were instructed to avoid swallowing for the duration of the retention period.

**Randomization, Allocation Concealment, and Implementation**

The random allocation sequence was generated using a computer-based random number generator. To ensure balanced group sizes over time, we employed a block randomization procedure with randomly varying block sizes of 3 and 6. Participants were randomly assigned to one of the three treatment groups detailed below.

| Group | Capsule (90 min pre-task) | Spray (45 min pre-task) |
| --- | --- | --- |
| 1 | Losartan | Placebo |
| 2 | Placebo | Placebo |
| 3 | Placebo | Vasopressin |

To conceal the allocation sequence and ensure double-blinding, an independent researcher, who was not involved in participant recruitment, data collection, or analysis, prepared all medications. The specific blinding procedure was as follows: All capsules were identical in appearance and dispensed in containers labeled with a unique code. All nasal sprays used identical devices and were marked to indicate the code. The researcher managed the allocation list and was solely responsible for assigning codes to the medications according to the randomization sequence. The research staff who enrolled participants and administered the treatments received only these pre-coded medications and had no access to the random sequence or the master list. They were only aware of the code for each participant but remained fully blinded to the corresponding treatment group assignment. This system successfully blinded all participants and research staff to group assignments, guaranteed that the group assignment was fully concealed until all data collection was complete and the database was locked, and was achieved through the code system with the use of visually identical capsules and matched placebos.

All interventions were administered by trained research staff according to a standardized protocol at a university laboratory at the University of Electronic Science and Technology of China (UESTC), Chengdu, China. Additionally, to ensure participant safety, research staff remained with participants after drug administration, and a dedicated safety officer was on call. All participants were instructed to report any discomfort immediately.

**Recruitment and Follow-up**

Dates of Recruitment: Participant recruitment and data collection for this trial took place from May 2024 to October 2024.

Follow-up for Outcomes: As this was a single-session laboratory study, all primary and secondary outcome measures (behavioral and pupillometry data) were collected during the experimental session. Therefore, no long-term follow-up was conducted for these efficacy outcomes. However, to monitor potential adverse effects, a follow-up text message was sent to all participants 24-48 hours after the experiment to inquire about any delayed discomfort or adverse events. No such events were reported.

The trial ended as planned, after the pre-determined target sample size was recruited.

**Stimulus Preparation and Presentation**

Stimulus images were normalized to a size of 400 × 250 pixels against a uniform grey background using Adobe Photoshop. The experiment was programmed and presented using Psychtoolbox-3 running under MATLAB (R2018b).

**Data Quality Control and Temporal Normalization**

To ensure the validity of our pupillometry analysis, trials were subjected to predefined quality control criteria prior to inclusion. These criteria were designed to remove trials with fundamental data integrity issues while aligning with established practices in pupillometry research [5]. The application of these criteria resulted in the exclusion of 9.65% of trials (1,506 trials), yielding 14,266 valid trials for analysis. This exclusion rate is well within the expected and acceptable range of 10–20% for pupillometry studies [6,7]. Critically, the number of excluded trials did not differ between treatment groups (*χ*²(2) = 0.46, *p* = .53), arguing against any biasing effects of the exclusion criteria on our results.

Trials were excluded based on the following principled criteria:

1. **Missing Behavioral Responses:** Trials without a recorded behavioral response were excluded, as they preclude the analysis of time-to-collision judgments.
2. **Insufficient Valid Data Points:** Trials with fewer than 10 overall valid data points, or fewer than 3 valid points within any predefined analysis phase (baseline, stimulus presentation, or imagination), were excluded. Given our 100 Hz sampling rate, the phase-specific requirement of 3 points translates to a lenient threshold of only 30 ms of valid data. This ensures we selectively excluded only trials with extreme artifacts (e.g., complete signal loss from prolonged blinks) while retaining the majority of data [8,9].
3. **Short Trial Duration (< 2300 ms)**: This criterion was informed by the minimum data requirement for our event-locked and time-normalized analyses. The calculation is as follows: 300 ms (baseline) + 1000 ms (stimulus presentation) + 1000 ms (minimum imagination period) = 2300 ms. This 1000 ms post-stimulus window was critical to cleanly extract the late imagination epoch (the final 500 ms before response) without truncation from an early response. Including shorter trials would introduce inconsistency and potential bias.
4. **Poor Signal Integrity:** Signal integrity was verified through temporal continuity checks and autocorrelation analysis to identify and eliminate trials with pervasive, uncorrectable high-frequency noise, ensuring results are not driven by artifact-laden data [5].

For subsequent analysis of the variable-duration imagination periods, we implemented a piecewise linear time warping procedure to normalize temporal dynamics across trials, a common approach for handling temporal misalignment in functional data [10]. The approach preserved the functional segregation between stimulus processing and imagination phases by mapping the fixed stimulus presentation epoch (300-1300 ms) to the normalized interval [0, 0.5], while the variable imagination epoch (from 1300 ms to response) was mapped to [0.5, 1.0]. Each normalized epoch was resampled at 100 equidistant points, creating a standardized 200-point representation per trial that maintained the relative temporal structure within phases while enabling direct comparison across trials. Finally, the normalized time series were smoothed using cubic B-spline functions (order 3, 4 basis components) to reduce high-frequency noise while preserving authentic temporal dynamics in the pupillary response [11,12].

**Results**

**Pre-specified Analysis on Collision Time**

Analysis of judged time-to-collision (jTTC) showed that threatening stimuli induced shorter time estimations compared to non-threatening ones (*F* = 30.78, *p* < .001, $\text{η}_{\text{p}}^{\text{2}}$ = 0.23), with females showing overall shorter estimations (*F* = 6.71, *p* = .011, $\text{η}_{\text{p}}^{\text{2}}$ = 0.06). Higher approaching velocities predictably led to shorter estimations (*F* = 51.17, *p* < .001, $\text{η}_{\text{p}}^{\text{2}}$ = 0.67).

These main effects were accompanied by two significant interactions. The PSV × IsThreaten interaction (*F* = 9.26, *p* < .001, $\text{η}_{\text{p}}^{\text{2}}$ = 0.27) revealed that threat-induced temporal compression was present across velocities V1-V4 but disappeared at V5 (fastest velocity). This finding reflects successful threat induction via looming and an affective (threat) modulation of the effect. Similarly, the Sex × PSV interaction (*F* = 5.06, *p* < .001, $\text{η}_{\text{p}}^{\text{2}}$ = 0.17) showed that females exhibited shorter time estimations than males across velocities V1-V4, while this sex difference disappearing at V5.

While no overall treatment effect (LT, PLC, AVP) was found (*F* = 2.07, *p* = .13), planned comparisons between AVP and PLC revealed a main effect of treatment, with AVP significantly prolonging time estimations (*F* = 3.99, *p* = .049, $\text{η}_{\text{p}}^{\text{2}}$ = 0.05). No significant difference was observed between LT and PLC.

We finally conducted the specified contrast with weights (-1, 2, -1) on the initial ANOVA model, which did not yield a significant effect (*F* = 2.51, *p* = .12), confirming the absence a unified effect of both drugs (combined) compared to placebo. This null finding is indeed informative. It suggests that the pharmacological actions of AVP and LT did not produce a consistent, unidirectional shift in time estimation relative to PLC. Instead, they may influence temporal perception in distinct and potentially opposing ways that are not captured by analyses of overall means.

**Functional Principal Components**

Functional Principal Component Analysis (FPCA) of pupillary dynamics revealed three dominant components (eigenfunctions) accounting for 87.51% of total variance. As shown in **S1 Fig**, their temporal weight functions displayed distinct patterns across the stimulus approach (0-0.5 s) and post-stimulus imagination (0.5-1.0 s) phases: PC1 (62.7%) showed positive weights that rose during stimulus approach and plateaued during imagination; PC2 (17.6%) transitioned from negative weights during approach to positive weights during imagination; and PC3 (7.3%) exhibited a positive-negative-positive oscillation spanning both phases.

**(1). Effects of Experimental Conditions on Principal Components**

Mixed ANOVAs on the principal components (PC1-3) revealed distinct effects of experimental conditions (**S2 Fig**).

All components showed significant PSV main effects (PC1: *F* = 105, *p* < .001, $\text{η}_{\text{p}}^{\text{2}}$ =.40; PC2: *F* = 105, *p* < .001, $\text{η}_{\text{p}}^{\text{2}}$ =.31; PC3: *F* = 7.32, *p* = .005, $\text{η}_{\text{p}}^{\text{2}}$ =0.63), each with distinct patterns: extreme speeds (V1, V5) elicited higher PC1 scores but lower PC3 scores than medium speeds (V2-V4), while PC2 showed progressively increased scores with speed (**S2 Fig. C**).

For PC1 (sustained dilation pattern), we found a significant Treatment × Sex × Isthreaten interaction (*F* = 3.37, *p* = .023, $\text{η}_{\text{p}}^{\text{2}}$ =0.69). Females showed treatment-dependent responses to threat: under non-threatening conditions, the PLC group exhibited higher PC1 scores than drug groups (*p*s < .01), while under threatening conditions, both PLC and AVP groups showed higher scores than the LT group (*p*s < .05); Males showed no treatment effects across conditions (*p*s > .48) (**S2 Fig. A1, A2**).

PC2 (biphasic pattern) demonstrated significant main effects of Treatment (*F* = 7.82, *p* < .001, $\text{η}_{\text{p}}^{\text{2}}$ = 0.30) and Isthreaten (*F* = 9.76, *p* = .002, $\text{η}_{\text{p}}^{\text{2}}$ = 0.24). The AVP group showed higher PC2 scores compared to other groups (AVP vs PLC: *p* =.006; AVP vs LT: *p* = .025), whereas LT-PLC showed no difference (*p* = .53). Threatening stimuli elicited higher scores than non-threatening ones (*p* < .001). A significant Treatment × Isthreaten interaction revealed greater responses to threatening versus non-threatening stimuli in both drug groups (*p*s < .01), but not in PLC (*p* = .45) (**S2 Fig. B**).

PC3 (triphasic pattern) showed a significant main effect of Isthreaten (*F* = 8.12, *p* = .005, $\text{η}_{\text{p}}^{\text{2}}$ = 0.22), with threatening stimuli eliciting higher scores than non-threatening ones.

**(2). Relationships Between Principal Components and Individual Difference Variables**

We examined correlations between pupillary response components and individual differences in anxiety changes (Post-Pre State Anxiety scores) and judged time-to-collision (jTTC).

The most notable finding was a moderate negative correlation between PC2 (biphasic pattern) and anxiety change scores (*r* = -0.302, *p* = .001) (**S2 Fig. D**). Further analysis comparing high-anxiety (top 20%) versus low-anxiety (bottom 20%) groups revealed significantly lower PC2 scores in the high-anxiety group (M = -0.025, SD = 0.035) compared to the low-anxiety group (M = 0.010, SD = 0.03) (*t* = 3.22, *p* = .003, Cohen’s *d* = 1.09). This suggests that individuals with increased anxiety showed stronger pupil dilation during stimulus presentation but enhanced constriction after stimulus offset and provides a behavioral link of the PC2 to subjective anxiety.

For behavioral responses, PC3 (triphasic pattern) showed a significant positive correlation with jTTC (*r* = 0.263, *p* = .004), indicating that slower responders exhibited a distinctive pattern of weakened dilation followed by enhanced constriction-dilation cycles. PC1 showed a marginal negative trend with jTTC (*r* = -0.169, *p* = .069), while PC2’s correlation with jTTC was non-significant (*r* = 0.147, *p* = .114) (**S2 Fig. E**).

**(3). Associations Between FPCA-derived Components and Phase-specific Pupillary Features**

PC1 showed strong positive correlations with pupil dilation state (*r* = 0.615) and change rate (*r* = 0.650) during stimulus presentation, while negatively correlating with baseline pupil diameter (*r* = -0.177), suggesting greater dilation potential in participants with smaller baseline pupils.

PC2 demonstrated strong positive correlations with post-stimulus pupil dilation state (*r* = 0.668) and change rate (*r* = 0.603), while showing a moderate negative correlation with during-stimulus pupil state (*r* = -0.352), capturing the inhibition-to-activation transition.

PC3 exhibited strong negative correlation with during-stimulus change rate (*r* = -0.580) and a moderate positive correlation with post-stimulus change rate (*r* = 0.337), reflecting temporal dynamic regulation (**S2 Fig. F**). A summary and interpretation of the principal components (fPCs) results are provided in Table 1 in the main text.

**Markov Chain Analysis**

**Steady State Distribution**

Markov chain analysis revealed stable probability distributions of pupillary response patterns across experimental conditions. We conducted chi-square tests and residual analyses on the main effects of Treatment, Sex, Isthreaten, and PSV, with the following results:

Treatment(*χ*²(14) = 333.17, *p* < .001, Cramer’s *V* = 0.107). Residual analysis revealed distinct pattern preferences across treatment conditions. Pattern E1 (PC1+, PC2-, PC3-), characterized by sustained pupil dilation with initial dilation followed by constriction, emerged as a key discriminator between PLC and AVP conditions (PLC: *z* = 8.55; AVP: *z* = -9.86). Pattern L3 (PC1-, PC2+, PC3+), showing sustained constriction with enhanced late activation, reached higher equilibrium states in both AVP and LT groups (*z* = 4.83 and 3.51, respectively) compared to PLC (*z* = -8.18). Pattern L2 (PC1-, PC2-, PC3+) differentiated AVP from LT, showing elevated equilibrium probability in AVP (z = 3.60) but reduced in LT (*z* = -2.72).

Sex(*χ*²(7) = 250.84, *p* < .001, Cramer’s *V* = 0.133). Pattern E3 was significantly higher than expected in females but significantly lower than expected in males. Patterns L1 and L2 were significantly higher than expected in males (L1: *z* = 6.86; L2: *z* = 2.59) but significantly lower than expected in females (L1: *z* = -7.27; L2: *z* = -2.74).

Isthreaten (*χ*²(7) = 73.04, p < .001, Cramer’s *V* = 0.072). Under non-threatening conditions, the system tended toward E1 (*z* = 3.06) at equilibrium, while threatening stimuli shifted the stable state toward E2 patterns (*z* = 4.21). Pattern E2 (PC1+, PC2+, PC3+), exhibiting early pupil dilation with enhanced activation, represented a distinct stable state specifically associated with threat processing.

PSV (*χ*²(28) = 549.29, *p* < .001, Cramer’s *V* = 0.098). Residual analysis revealed systematic velocity-dependent patterns. Slower velocities (V1-V2) predominantly evoked late response patterns (L1, L2), with significant positive residuals for L1 (V1: *z* = 5.62; V2: *z* = 4.30) and L2 (V1: *z* = 5.39; V2: *z* = 3.89), while inhibiting early patterns, especially E2 (V1: *z* = -3.68; V2: *z* = -5.28) and E3 (V1: *z* = -6.70). Moderate velocity (V3) showed a transitional state with mixed responses, featuring positive residuals for E3 (*z* = 2.29) and L3 (*z* = 3.57) but negative for E1 (*z* = -3.93). Faster velocities (V4-V5) strongly triggered early responses, particularly E3 for V4 (*z* = 4.56) and both E1 (*z* = 6.02) and E2 (*z* = 7.87) for V5, while significantly inhibiting late patterns across L1-L4 clusters. This velocity gradient in pupillary responses likely reflects differential processing of approach speed as a threat cue, with faster approaches demanding more immediate attentional and autonomic resources.

**Dynamics Transition**

We analyzed transition probabilities using chi-square tests and residual analysis for overall effects, and bootstrap permutation tests (1000 iterations) with FDR correction for specific transitions. This combined approach allowed us to identify statistically significant overall effects while establishing reliable confidence intervals for individual transition probabilities.

**Treatment**

Response patterns showed high self-transition probabilities (0.451-0.630) across all conditions, indicating stable response strategies. Self-transition probabilities varied by treatment: PLC group showed highest stability in E1(0.628) and L1(0.616); LT group exhibited comparable stability in E3(0.609) and E1(0.607); while AVP group was dominated by E3(0.630) and L3(0.583) patterns. All self-transitions were statistically significant (*p*s<.001).

Between-state transitions also showed treatment-specific characteristics. PLC group featured transitions toward E1 (E4→E1: 0.177; E3→E1: 0.175), LT group showed prominent E4→E1(0.166) and E2→E3(0.145) transitions, while AVP group was characterized by E1→E3(0.171) transitions (all *p*s<.001; 95% CI in Table S2).

FDR-corrected group comparisons revealed distinct treatment effects on transition patterns PLC group showed significantly increased probabilities in transitions targeting E1, including E1→E1(*z* = 2.39), E2→E1(*z* = 2.51), E3→E1(*z* = 4.65), and E4→E1(*z* = 2.49). In contrast, AVP group showed reduced transitions toward E1 (E1→E1: *z* = -4.30; E2→E1: *z* = -2.83; E4→E1: *z* = -3.40) but increased transitions involving L2 and L3 patterns (E4→L2: *z* = 2.92; L3→L3: *z* = 2.83; L1→L3: *z* = 2.54). LT group specifically showed reduced transitions toward L2 pattern (E2→L2: *z* = -2.32; E4→L2: *z* = -1.99).

These results indicate that PLC enhances transitions toward E1 pattern, whereas AVP promotes transitions toward E3, L2, and L3 patterns while reducing E1-directed transitions. Notably, LT selectively inhibits transitions toward L2 pattern (PC1+, PC2+, PC3+), reflecting suppressed activation across all three principal components.

**Isthreaten**

For threat effects, E3→E2 transitions were significantly modulated by Isthreaten (*χ*²(1) = 14.95, *p*-adj = 0.007), with increased probability under threatening conditions (*z*=2.54) and decreased probability under non-threatening conditions (*z*=-2.62).

**Sex**

Sex significantly influenced pupillary response patterns (*χ*²(7) = 250.84, *p* < .001). Male participants showed higher probabilities for transitions involving late response(L-pattern), particularly with elevated L1 (*z* = 6.86, *p* < .001), L2 (*z* = 2.59, *p* < .01), and L4 (*z* = 2.29, *p* < .05) representations. In contrast, female participants exhibited significantly increased E3 pattern (*z* = 7.60, *p* < .001) with corresponding decreases in late response patterns: L1 (*z* = -7.27, *p* < .001), L2 (*z* = -2.74, *p* < .01), and L4 (*z* = -2.42, *p* < .05).

These sex differences were further reflected in transition probabilities, where males showed higher self-transition stability for L1→L1 (0.611) compared to females (0.547), while females exhibited enhanced E3→E3 stability (0.617 vs. 0.575 in males). Additionally, females demonstrated more frequent transitions from E1→E3 (0.158) and E2→E3 (0.158) compared to males, suggesting sex-specific differences in pupillary response modulation.

**Physical Stimulus Velocity (PSV)**

PSV significantly impacted pupillary dynamics (*χ*²(7) = 73.04, *p* < .001). High-PSV participants showed distinct transition characteristics compared to low-PSV participants. Participants in high-PSV trials exhibited increased self-transition stability in E1→E1 (0.609) and E2→E2 (0.565) patterns, while low-PSV demonstrated enhanced E3→E3 stability (0.631).

Notable differences appeared in steady-state distributions, with high-PSV participants showing greater long-term prevalence of E1 (0.237) and E2 (0.169) patterns, while low-PSV participants showed enhanced E3 representation. This suggests that approaching velocity influences the preferred pupillary response mode, with high-PSV individuals favoring E1 (PC1+, PC2-, PC3-) patterns that reflect specific components of physiological arousal.

**Discussion**

**Sex Differences in Patterns**

Pattern analysis provides a unifying multi-level account of these differences by identifying distinct sex-biased neurocognitive states:

**Male-biased** **Profile L1** (+, -, +) is characterized by high sustained vigilance (PC1+) and high cognitive dynamics (PC3+), but low proactive threat preparation (PC2-). This cognitive profile is reflected in distinct pupillary dynamics, showing an initial expansion followed by a pronounced contraction, which aligns with a strategy of generalized environmental monitoring. The L-subgroup (late-response) manifests as longer judged time-to-collision (jTTC), indicating a tendency toward temporal overestimation and delayed responding. Overall, males maintain a high baseline readiness but invest less in specific, sustained threat simulation once a stimulus is categorized, consistent with their lack of post-stimulus threat discrimination. This pattern may reflect a tonic state of exploratory vigilance optimized for broad monitoring rather than deep threat-specific processing [13], which behaviorally manifests as a less precautionary response strategy.

**Female-biased Profile E3** (-, +, +) presents a contrasting pattern: low sustained vigilance (PC1-), high proactive preparation (PC2+) and high internal simulation (PC3+), which marked by pupillary constriction during stimulus approach that transitions to dilation after its offset, suggesting a strategy of targeted resource allocation. The E-subgroup (early-response) was associated with shorter judged TTC, indicating a tendency toward temporal underestimation and accelerated responding. Females appear to suppress initial generalized vigilance to preferentially engage in sustained, internal threat modeling and preparation after stimulus disappearance, directly explaining their robust post-stimulus threat differentiation and behavioral bias towards earlier responses. This suggests a phasic, threat-specific resource investment strategy geared towards detailed evaluation of relevant dangers [14], which behaviorally manifests as the previously identified precautionary policy of early action.

In summary, the male-biased strategy (Profile L1) manifests as a state of broad, shallow monitoring that maintains generalized environmental readiness at the cost of detailed threat evaluation, resulting in delayed defensive responses. Conversely, the female-biased strategy (Profile E3) facilitates a state of sustained, targeted internal modeling of threats after stimulus offset, which supports superior threat discrimination and promotes the precautionary action of early responses. This functional dissociation provides a parsimonious model that directly links sex-specific neurocognitive states to their distinct behavioral phenotypes.

**Note**

**Refinements to the Analytical Approach**

The pre-specified confirmatory analyses for questionnaire and behavioral data were conducted as planned, and their full results are reported in the preceding section of this supplement. The initial ANOVA successfully identified key main effects and interactions regarding threat and velocity on time perception.

During a deeper investigation of the data, we recognized that the relationship between objective and subjective time-to-collision was inherently nonlinear. To move beyond the mean-based comparisons of ANOVA and to more precisely characterize the computational nature of this temporal distortion, we employed nonlinear modeling as the primary analytical framework for behavior in the main text. This refined approach revealed a critical, fine-grained insight that was not accessible to the pre-specified ANOVA: the magnitude of threat-induced temporal compression systematically varied as a function of the objective time-to-collision (aTTC).

Similarly, for the pupillometry data, we adopted an exploratory analytical framework using Functional Principal Component Analysis (FPCA) combined with clustering and Markov Chain Analysis. This methodology was chosen to objectively identify and characterize the distinct, dynamic temporal patterns in the pupillary response that are obscured by standard averaged analyses.

The findings from these advanced analyses are presented in the main manuscript because they provide a more comprehensive and computationally precise characterization of the underlying processes. The results of the pre-specified ANOVA are reported above in full to ensure complete transparency and to demonstrate the robustness of the core threat effect.

The adoption of these refined analytical approaches constitutes the important change to the trial protocol after its commencement.

**Reference**

1. Gao Z, Ma X, Zhou X, Xin F, Gao S, Kou J, et al. Oxytocin reduces the attractiveness of silver-tongued men for women during mid-cycle. Front Neurosci. 2022;16. doi:10.3389/fnins.2022.760695

2. Garver-Apgar CE, Gangestad SW, Thornhill R. Hormonal correlates of women’s mid-cycle preference for the scent of symmetry. Evolution and Human Behavior. 2008;29: 223–232. doi:10.1016/j.evolhumbehav.2007.12.007

3. Kou J, Lan C, Zhang Y, Wang Q, Zhou F, Zhao Z, et al. In the nose or on the tongue? Contrasting motivational effects of oral and intranasal oxytocin on arousal and reward during social processing. Transl Psychiatry. 2021;11: 94. doi:10.1038/s41398-021-01241-w

4. Zhuang Q, Zheng X, Yao S, Zhao W, Becker B, Xu X, et al. Oral administration of oxytocin, like intranasal administration, decreases top-down social attention. Int J Neuropsychopharmacol. 2022;25: 912–923. doi:10.1093/ijnp/pyac059

5. Mathôt S, Fabius J, Van Heusden E, Van der Stigchel S. Safe and sensible preprocessing and baseline correction of pupil-size data. Behav Res. 2018;50: 94–106. doi:10.3758/s13428-017-1007-2

6. Hayes TR, Petrov AA. Mapping and correcting the influence of gaze position on pupil size measurements. Behav Res. 2016;48: 510–527. doi:10.3758/s13428-015-0588-x

7. Winn B, Whitaker D, Elliott DB, Phillips NJ. Factors affecting light-adapted pupil size in normal human subjects. Invest Ophthalmol Vis Sci. 1994;35: 1132–1137.

8. Hershman R, Henik A, Cohen N. A novel blink detection method based on pupillometry noise. Behav Res. 2018;50: 107–114. doi:10.3758/s13428-017-1008-1

9. Kret ME, De Dreu CKW. The power of pupil size in establishing trust and reciprocity. J Exp Psychol Gen. 2019;148: 1299–1311. doi:10.1037/xge0000508

10. Ramsay JO, Silverman BW. Principal components analysis for functional data. Functional Data Analysis. New York, NY: Springer New York; 1997. pp. 85–109. doi:10.1007/978-1-4757-7107-7_6

11. Dan EL, Dînşoreanu M, Mureşan RC. Accuracy of six interpolation methods applied on pupil diameter data. 2020 IEEE International Conference on Automation, Quality and Testing, Robotics (AQTR). 2020. pp. 1–5. doi:10.1109/AQTR49680.2020.9129915

12. Eilers PHC, Marx BD. Flexible smoothing with B-splines and penalties. Statistical Science. 1996;11: 89–121. doi:10.1214/ss/1038425655

13. Aston-Jones G, Cohen JD. AN INTEGRATIVE THEORY OF LOCUS COERULEUS-NOREPINEPHRINE FUNCTION: Adaptive gain and optimal performance. Annual Review of Neuroscience. 2005;28: 403–450. doi:10.1146/annurev.neuro.28.061604.135709

14. Blanchard DC, Griebel G, Pobbe R, Blanchard RJ. Risk assessment as an evolved threat detection and analysis process. Neuroscience & Biobehavioral Reviews. 2011;35: 991–998. doi:10.1016/j.neubiorev.2010.10.016
