# Supplementary material for: Vasopressin and angiotensin II pathways differentially modulate human fear response dynamics to looming threats
Source: PLoS Biol. 2026 Feb 24;24(2):e3003668. doi: 10.1371/journal.pbio.3003668 (PMC12978571; doi:10.1371/journal.pbio.3003668)
Supplement: S1 File — (PDF) [file pbio.3003668.s013.pdf]

Title

Vasopressin and Angiotensin II Modulation of Human Looming-Threat Processing: A Randomized, Double-Blind, Placebo-Controlled Mechanistic Trial in Healthy Volunteers

1. Background and Rationale

Primitive defensive responses to looming threats are evolutionarily conserved mechanisms essential for survival. Pharmacological and behavioral evidence implicates two neuropeptide systems in modulating the functional dynamics of these defensive states:

- **Arginine vasopressin (AVP)**, which is hypothesized to promote a state of hypervigilance and heighten physiological arousal, thereby facilitating rapid defensive mobilization and anxiety-related biases in threat processing.
- **The renin–angiotensin system (RAS)**, where angiotensin II type 1 receptor (AT1R) blockade (e.g., via losartan) has been shown to dampen excessive autonomic reactivity and enhance the regulation of defensive responses, suggesting a potential stabilizing role in threat evaluation.

However, human evidence on how these systems shape the temporal and computational dynamics of looming threat processing remains scarce. Looming paradigms allow for the precise manipulation of threat characteristics (e.g., approach speed), while pupillometry serves as a high-resolution, non-invasive proxy for locus coeruleus–norepinephrine (LC-NE) activity, reflecting real-time shifts in central arousal and cognitive effort.

The present pharmacological eye-tracking administering oral AVP or the AT1R blocker losartan versus placebo was designed to:

1. Dissect how AVP and AT1R blockade modulate time-to-collision (TTC) perception during looming threats.
2. Characterize treatment-specific profiles of pupillary threat dynamics, including during visually occluded, imagined threat continuation.
3. Identify integrated behavioral–physiological "phenotypes" and their state-transition dynamics under each pharmacological condition.

The study was registered at ClinicalTrials.gov (NCT06329063, NCT06329076) prior to enrollment and approved by the local ethics committee of the University of Electronic Science and Technology of China (UESTC, approval number: 106142024050929462).

2. Objectives and Hypotheses

2.1 Primary Objective

To determine how AVP and losartan, relative to placebo, modulate subjective TTC estimation and associated pupillary dynamics during a looming- threat paradigm in healthy adults.

2.2 Secondary Objectives

1. To test whether losartan exerts an anxiolytic effect at the state level compared with placebo, as indexed by changes in State- Trait Anxiety Inventory (STAI- state) scores from pre- to post- task.
2. To investigate whether the pharmacological effects on tonic and phasic pupillary responses are modulated by biological sex (i.e., potential sex-dependent dissociations) across the perception and imagination phases of threat processing.
3. To identify integrated behavioral–pupillary response profiles and characterize their temporal evolution and state-transition dynamics across trials.

2.3 Hypotheses

1. **AVP and Threat Amplification:** AVP administration is hypothesized to amplify defensive vigilance and sensory intake. Unlike acute panic which precipitates premature reactions, we predict AVP will induce a "freezing-like" state characterized by an overestimation of TTC. This reflects a temporal dilation effect where the heightened processing of the looming threat results in a subjective slowing of the external event, thereby delaying the predicted collision point. Furthermore, we expect AVP to elicit sustained high-arousal pupillary responses, particularly during the imagination phase, indicating prolonged cognitive processing and threat maintenance.
2. **Losartan and Anxiolytic Effects:** Losartan is hypothesized to exert an anxiolytic effect on looming perception. We predict it will attenuate the threat-induced time compression bias (leading to shorter TTC estimates) and dampen pupillary dilation, reflecting a downregulation of the physiological threat response.
3. **Temporal Dynamics and State Stability:** We hypothesize distinct modulation of arousal state stability by the two treatments. AVP is expected to stabilize high-vigilance states (reducing flexibility) to ensure sustained monitoring, whereas Losartan will promote more flexible threat-response sequences, facilitating a faster return to baseline arousal following threat exposure.

3. Trial Design

Randomized, double- blind, placebo- controlled, parallel- group, single- center psychopharmacological trial with three treatment arms (AVP, losartan, placebo) and a single experimental session per participant.

- **Allocation ratio:** 1:1:1.

- **Total sample size:** We aim to recruit a total of 90 healthy volunteers (30 participants per group). To ensure balanced sex distribution, each group will be stratified to include an equal number of males and females (15 males and 15 females per arm).

4. Study Population

4.1 Exclusion Criteria

1. History of hepatic, renal, gastrointestinal, respiratory, endocrine, neuropsychiatric, cardiovascular diseases.
2. A history of cardiac disease including arrhythmias; history of syncope or unexplained loss of consciousness; history of renal stones or renal failure; history of diabetes mellitus or diabetes insipidus.
3. History of or present renal artery stenosis (reduced blood flow to the kidneys).
4. History of hypersensitivity or allergic reaction to any medication or hormone, strong allergic reaction to food, or general tendency for allergies.
5. Known hypersensitivity to losartan or related agents (e.g. telmisartan), antidiuretic hormone or any other component of the formulation; known hypersensitivity to any other angiotensin II receptor antagonist.
6. Subjects with hypertension (BP > 140/90mmHg) or hypothension (BP < 90/60mmHg).
7. Current hyponatremia (low levels of sodium in the blood) (symptoms include nausea, fatigue and weakness or swelling of hands, feet or legs)
8. History of alcohol or drug abuse； smoker (≥ 10 cigarettes or ≥ 3 cigars or ≥ 3 pipes/day)
9. Blood donation (≤ 1 month prior to administration)
10. Take oral contraceptives or receive hormonal medications in the three months prior to the experiment.
11. Pregnant or breastfeeding.
12. Current disorder, including infections such as COVID, flu or cold.

4.2 Participant Pre-screening Procedures

- Prior to participation, all volunteers underwent an electrocardiogram (ECG) examination to rule out potential cardiovascular contraindications. To minimize potential confounds from psychoactive substances, participants were required to abstain from caffeine and alcohol for 24 hours prior to the testing session.
- For female participants, a urine-based pregnancy test was administered on the day of the experiment to exclude pregnancy. Additionally, the phase of their menstrual cycle was documented based on self-report to account for potential hormonal influences.

5. Interventions

5.1 Investigational Products

1. **AVP**: 20 IU arginine vasopressin administered via a spray-based oral delivery method (6 alternating 0.1-ml puffs; total 0.6 ml).
2. **Losartan (LT)**: 50 mg oral capsule, targeting AT1R blockade.
3. **Placebos**: Spray identical in composition and appearance to AVP spray but without active ingredient; Capsules identical in appearance to losartan capsule but containing no active ingredient.

5.2 Dosing Schedule and Timing

To align with pharmacokinetic profiles—peak effect ~45 min for AVP and ~90 min for losartan—the trial used a two-stage administration protocol:

- **Group 1 (LT):** 50 mg losartan capsule 90 min before task; placebo spray 45 min before task.
- **Group 2 (PLC):** Placebo capsule 90 min before task; placebo spray 45 min before task.
- **Group 3 (AVP):** Placebo capsule 90 min before task; 20 IU AVP spray 45 min before task.

This design ensured that, at task onset, participants in each active group were within the expected peak plasma window while maintaining blinding.

5.3 Blinding

To conceal the allocation sequence and ensure double-blinding, an independent researcher, who was not involved in participant recruitment, data collection, or analysis, prepared all medications. The specific blinding procedure was as follows: All capsules were identical in appearance and dispensed in containers labeled with a unique code. All nasal sprays used identical devices and were marked to indicate the code. The researcher managed the allocation list and was solely responsible for assigning codes to the medications according to the randomization sequence. The research staff who enrolled participants and administered the treatments received only these pre-coded medications and had no access to the random sequence or the master list. They were only aware of the code for each participant but remained fully blinded to the corresponding treatment group assignment. This system successfully blinded all participants and research staff to group assignments, guaranteed that the group assignment was fully concealed until all data collection was complete and the database was locked, and was achieved through the code system with the use of visually identical capsules and matched placebos.

6. Procedures

6.1 General Procedure

Upon arrival at the laboratory, participants underwent a screening process involving a review of their medical and psychiatric history to ensure eligibility. Following enrollment, participants completed a battery of baseline questionnaires, including the Positive and Negative Affect Schedule (PANAS), the State-Trait Anxiety Inventory (STAI, state and trait), the Liebowitz Social Anxiety Scale (LSAS), and the Animal Fear Questionnaire (AFQ).

After completing the questionnaires, participants rested for approximately 10 minutes to stabilize their physiological state. Baseline blood pressure (systolic and diastolic) and heart rate were then measured. Based on a pre-assigned randomization code, double-blind study personnel administered the first stage of the treatment: an opaque capsule containing either 50 mg losartan or a placebo.

Following a 45-minute waiting period, the second stage of treatment was administered: an oral spray containing either 20 IU AVP or a placebo. Following our validated protocol [1,2], each participant received six separate 0.1 ml puffs (total volume = 0.6 ml), with a 30-second retention period between puffs. The puffs were administered in an alternating fashion: three puffs were directed to the superior surface of the tongue and three to the inferior surface. Participants were instructed to avoid swallowing for the duration of the retention period.

After the spray administration, participants underwent a second 45-minute waiting period to reach the peak pharmacokinetic window for both agents. Immediately prior to the start of the experimental task (approximately 90 minutes after capsule ingestion and 45 minutes after spray administration), blood pressure and heart rate were re-assessed to monitor potential cardiovascular effects. Participants then entered the testing room to complete the experimental tasks. Upon conclusion of the tasks, a final measurement of blood pressure and heart rate was taken, followed by the completion of post-experiment questionnaires.

6.2 Experimental Task: Looming Threat Paradigm

The task combined **visual approach** and **imagination** phases.

- **Stimuli:** Color photographs of threatening animals (snakes, spiders) and non-threatening animals (butterflies, rabbits); 40 images per category.
- **Trial structure:**

  1. **Fixation cross:** Presented for 300 ms.
  2. **Presentation phase:** An image expands at a constant velocity and disappears after 1300 ms.
  3. **Imagination phase:** Participants imagine the continued approach of the now-hidden stimulus and press a key when they judge it would collide with them. The judged TTC (jTTC) is calculated as the total time elapsed from the onset of the looming stimulus (at the beginning of step 2) to the key press.
  4. **Feedback:** An auditory confirmation beep follows the response, succeeded by a variable inter-trial interval (1000–3000 ms).

- **Manipulations:** Five levels of actual TTC (aTTC: 3.0, 3.5, 4.0, 4.5, 5.0 s); Two initial stimulus sizes (20% or 30% of screen width); Four stimulus categories (threat vs non-threat).
- **Design:** Full factorial combination of aTTC (5) × category (4) × initial size (2) across 160 trials (two blocks). Participants first completed practice trials with silhouettes.
- **Pupillometry:** Pupil size from the right eye recorded at 2000 Hz using EyeLink 1000 Plus, with a 9-point calibration each block; data preprocessed to remove blinks/artifacts and down-sampled to 10-ms bins.

7. Outcomes

7.1 Primary Outcomes

Behavioral Outcome: The primary behavioral endpoint is the judged time-to-collision (jTTC) recorded on each trial. This millisecond-scale measure directly quantifies the subjective perception of looming threat and will be used to test for treatment-induced alterations in temporal estimation.

Pupillary Outcomes: Pupil diameter dynamics, analyzed as: (1) Mean pupil diameter within functionally defined time windows (e.g., perception, occlusion phases), and (2) Time-normalized pupil trajectories representing continuous arousal dynamics.

7.2 Secondary and Exploratory Outcomes

State Anxiety: Change in State-Trait Anxiety Inventory (STAI-State) scores from pre- to post-task.

Cardiovascular Monitoring: Systolic and diastolic blood pressure, and heart rate, measured at multiple time points to monitor for systemic physiological effects of the drug treatments.

Model-Based Decomposition of Time Perception: Parameters derived from fitting a power-law model (jTTC = $\alpha \cdot$ aTTC^β) to the behavioral data. The scaling (α) and compression (β) parameters will be analyzed to dissect potential drug effects on distinct components of temporal judgment (general bias vs. nonlinear distortion).

Integrated Behavioral-Physiological Phenotypes: Exploratory outcomes include latent response features representing dominant patterns of pupillary variability, multivariate cluster memberships defining distinct behavioral-physiological profiles, and dynamic state metrics (e.g., transition probabilities and stability indices) that quantify the temporal evolution and fluctuation of these response states across trials.

8. Sample Size Justification

An a priori power analysis (G*Power 3.1) for the primary mixed-design ANOVA (factors: Treatment [3], Sex [2], Threat [2], Velocity [5]; $f = 0.20$, $\alpha = 0.05$, power = 0.95) indicated a minimum sample size of 78. To ensure robust power and account for potential attrition, we will recruit a total of 90 participants (30 per group).

9. Randomization and Allocation Concealment

- Participants were randomized (stratified according to sex) in equal proportions to AVP, losartan, or placebo using computer-generated randomization lists.
- Allocation was implemented by staff not involved in data collection; treatment codes were held by a third party until completion of primary analyses.
- Capsules and sprays were visually indistinguishable across conditions to preserve blinding.

10. Data Collection, Management, and Quality Control

- Behavioral data (jTTC) and pupillary time series were logged digitally during the task.
- Pre-specified preprocessing steps:
    - Exclusion of trials with technical artifacts or incomplete responses.
    - Exclusion of trials with jTTC > 5 SD from an individual's mean.
    - Removal of pupillary values indicating blinks, loss of tracking, or physiologically implausible diameters; outliers > 5 SD removed and remaining data down-sampled to 10-ms bins.
- Time-warping procedures aligned trials to a normalized time axis (0–0.5 perception, 0.5–1 imagination) for FLMM and FPCA analyses.
- Anonymized data have been deposited in the Open Science Framework (OSF) and are openly available for verification and replication.

11. Statistical Analysis Plan

11.1 General Approach

Data analysis will be conducted using SPSS and Python. The significance level is set at $\alpha = 0.05$ (two-tailed). To control for the false discovery rate (FDR) across multiple testing, the Benjamini-Hochberg procedure will be applied.

11.2 Analysis of Behavioral Outcomes

- **Primary Analysis:** Treatment effects on judged time-to-collision (jTTC) will be assessed using Repeated Measures ANOVA, with Treatment, Sex, and Threat Condition as factors.
- **Psychophysical Modeling:** To dissect temporal perception mechanisms, we will fit psychophysical models (e.g., power-law functions: $\text{jTTC} = \alpha + \beta * \text{aTTC}$) to the behavioral data. The derived parameters (α and β) will be compared between treatment groups using LMMs.

11.3 Analysis of Pupillary Measures

- **Baseline & Phasic Responses:** Baseline pupil diameter and mean pupil diameter within event-locked phases will be analyzed using LMMs or Repeated Measures ANOVA, testing for main effects of Treatment and interactions with Sex and Stimulus conditions.

- **Pupil Trajectories:** To account for variable trial durations, pupillary time-series data will be time-normalized to a unified scale (e.g., 0 to 1) representing the progression of the looming phase. We will then analyze the continuous temporal evolution of pupil diameter using appropriate time-series analysis techniques (e.g., cluster-based permutation tests or mixed-effects modeling) to identify each treatment groups' arousal trajectories.

11.4 Exploratory Integrated Analyses

To identify potential treatment-specific phenotypes, we will employ multivariate, data-driven techniques:

- **Dimensionality Reduction:** Dominant features of pupillary variability will be extracted using functional decomposition methods (e.g., Functional PCA).
- **Clustering:** Trial-level behavioral and physiological features will be combined to identify distinct response profiles (clusters) using unsupervised learning algorithms.
- **State Dynamics:** The temporal evolution and stability of these response states across the experiment will be modeled using stochastic methods (e.g., Markov Chain Analysis) to quantify transition probabilities between states.

12. Safety Monitoring

- Blood pressure and heart rate monitored at baseline, peak drug effect, and post-task to detect cardiovascular changes.
- Adverse events (AEs) solicited at each contact and spontaneously reported AEs documented.
- Prespecified criteria for discontinuation included clinically significant blood pressure elevation, allergic reactions, or participant request. No serious adverse events were expected based on doses established in prior work.

13. Ethical Considerations and Dissemination

The protocol was reviewed and approved by the institutional ethics committee of UESTC and conducted in accordance with the Declaration of Helsinki. All participants provided written informed consent and received monetary compensation.

Trial results are to be disseminated through peer-reviewed publications and presentations at scientific conferences. De-identified datasets and analytic code will be made available in public repositories in line with journal and funder policies.

Reference

1. Kou J, Lan C, Zhang Y, Wang Q, Zhou F, Zhao Z, et al. In the nose or on the tongue? Contrasting motivational effects of oral and intranasal oxytocin on arousal and reward during social processing. Transl Psychiatry. 2021;11: 94. doi:10.1038/s41398-021-01241-w

2. Zhuang Q, Zheng X, Yao S, Zhao W, Becker B, Xu X, et al. Oral administration of oxytocin, like intranasal administration, decreases top-down social attention. Int J Neuropsychopharmacol. 2022;25: 912–923. doi:10.1093/ijnp/pyac059
