# Supplementary material for: Vasopressin and angiotensin II pathways differentially modulate human fear response dynamics to looming threats
Source: PLoS Biol. 2026 Feb 24;24(2):e3003668. doi: 10.1371/journal.pbio.3003668 (PMC12978571; doi:10.1371/journal.pbio.3003668)
Supplement: S2 File — (PDF) [file pbio.3003668.s014.pdf]

电子科技大学
科研项目伦理审查申请表
（快速审查）

（涉人体研究）

项目名称：氯沙坦和加压素对自上而下和自下而上控制的影响

项目负责人：Benjamin Becker 教授 [Signature redacted for privacy]

申请学院（公章）：生命科学与技术学院

研究领域：认知神经科学

申请阶段：☐项目申报 ☑论文发表

申请日期：2024 年 5 月 9 日

受理编号：1061420240509 29462

涉人体科研项目伦理审查申请表填写说明

（持续完善）

1. 提交材料时，科研项目伦理审查申请表（以下简称“申请表”）均需要如实填写，在对应框内☑，未作特殊要求的均为必填项；
2. 请勿随意更改申请表格式，需注意签章页勿跨页；
3. 涉及相关实体实验、个人数据隐私等情况，需要提交受试知情同意书（受试者签字确认）（附件 1）
4. 需要提交具体研究相关计划及可能存在的伦理问题说明（附件 2）；
5. 若我校为参与单位需要提供牵头单位伦理审查批件或情况说明（附件 3）；
6. 2021 年 9 月份以后，实验人员需要提供相关培训时长证明（附件 4）或从业人员岗位证书。若不涉及相关实体实验，可提供关于科研伦理相关知识的自学情况；
7. 研究计划需脱密后提供申报书/计划书/研究实验内容概述（附件 5）；
8. 如果涉及到生物样本出境检测的需要提供政府的相关审批件；
9. 若无需提供相关附件，则填写“无”；
10. 伦理委员会办公室将申请表收集齐全后组织专家会评，评审后返回审查结果；
11. 申报材料按照申报计划在科研管理系统中填报。

科研项目伦理审查申请表

项目名称：氯沙坦和加压素对自上而下和自下而上控制的影响	
申请日期：2024年5月9日	受试对象：人体

主要研究者信息	项目负责人姓名/职称：Benjamin Becker 教授	国籍：德国
	联系电话：15828121558	邮箱：ben_becker@gmx.de
	二级申报单位：生命科学与技术学院	（初步审查并加盖公章）

简要描述研究目的以及针对研究对象开展的研究方法和内容：

本研究的主要目的是调查加压素（20IU）和氯沙坦（50mg）是否会影响个体在社会情绪扫视/反扫视眼动追踪范式中的注意力控制以及个体对迫在眉睫的恐惧刺激的注意检测。

加压素是一种内源性神经肽，其分泌受到血管紧张素Ⅱ的调节；氯沙坦则是一种血管紧张素Ⅱ1型受体拮抗剂。二者均被证实可以调节个体社会认知和情感加工，但目前还尚不清楚加压素和氯沙坦对威胁性刺激的注意力分配的调节作用，包括刺激驱动的自下而上的注意控制和目标导向的自上而下注意控制。

本研究拟招募90名在校健康大学生，采用双盲对照临床实验范式，将被试随机分配到加压素组、氯沙坦组和安慰剂组。治疗后90分钟，受试者首先完成社会情绪扫视/反扫视眼动追踪任务，该范式使用社交以及非社交刺激来探索加压素或氯沙坦对社交和情绪特异性影响。随后受试者完成恐惧逼近任务，该任务使用威胁和非威胁刺激来探索药物对注意和反应时的影响。

可能存在的伦理风险：本研究拟用的口喷加压素主要成分是加压素、生理盐水和甘油。氯沙坦钾片是经国家食品药品监督管理局（国药准字H20000371）批准的，在全球广泛使用并且被认为是最安全，最有效的降压药物。**实验设备及实验过程均安全可靠，对人体无害。涉及的个人隐私全程保密。**

项目来源	☐ 国际合作项目（请说明：　　　　　　　　　）
	☑ 国家级基金项目（请说明：外国优秀青年学者研究基金项目 32250610208）
	☐ 省部级科研项目（请说明：　　　　　　　　　）
	☐ 其他（请填写：　　　　　　　　　）
本人承诺	我承诺对该实验方案的真实性负责。针对项目涉及的各类安全风险，严格执行国家相关法律法规及学校有关规定，落实相应的安全防范措施，确保在安全条件开展相关实验[Signature redacted for privacy]理委员会及社会各界的监督。<br>项目负责人签字 2024.5.9
审查意见	同意该实验方案。<br>校伦理委员会主任签字[Signature redacted for privacy]（校伦理委员会签章）

备注：此页勿跨页，请勿改变格式

一、涉及人的科研项目填写

本项目计划招募受试者人数/总人数： 90	预期试验起止时间：2023/5-2024/8
研究过程可能对受试者造成严重伤害	□ 是 ☑ 否
多中心研究	☑ 否<br>□ 是： □ 国际多中心牵头单位 □国际多中心参与单位<br>□ 国内多中心牵头单位 □国内多中心参与单位<br>（若我校为参与单位需要提供牵头单位伦理审查批件）
是否涉及到生物样本出境检测	☑ 否 □ 是→ 样本类型：<br>检测项目：<br>实验室名称：<br>地址：<br>是否涉及人类遗传资源检测：□ 否 □ 是
研 究 类 型	☑ 干预性研究 □非干预性研究
研 究 对 象	☑ 正常人 □病人 | 受试者年龄范围 | 18 岁 — 30 岁
弱 势 群 体	□精神疾病 □病入膏肓者 □孕妇 □文盲 □未成年人<br>□认知损伤者 ☑ 不涉及<br>□其他，具体说明：
要求排除对象	□无 □男性 □女性 ☑ 未成年人 □其他____________
要求具备的特殊条件	□重症监护 □手术 □儿童重症监护 □义肢<br>□静脉输注 □基因治疗 □计算机断层扫描 □妇科<br>□管制药品（麻醉药/精神药） □隔离区 ☑ 无<br>□其他（请具体说明）
	☑ 1. 受试知情书及同意书<br>☑ 2. 可能存在伦理问题说明<br>☑ 3. 牵头单位伦理审查批件或说明<br>☑ 4. 培训时长证明<br>☑ 5. 申报书或计划书完整版<br>□ 6. 其他 （勾选并在后附页）
主要研究者声明	此次试验的受试来源：在校成年大学生，招募方式：线上招募，履行知情同意程序以及经济补偿，保护被试人隐私。本人保证以上信息真实准确，并对该试验全过程中的质量保证负责，确保该临床试验数据真实可靠，操作规范，符合伦理学要求，并承诺本研究团队人员与该项目无利益冲突。如发生违规而造成的后果，由本人承担。<br>主要研究者[Signature redacted for privacy] 2024 年 5 月 9 日

附件 1

受试知情书（涉人实验提供）

尊敬的受试者：

您好！我们是电子科技大学神经信息教育部重点实验室的研究人员，欢迎您参加本实验。请务必认真阅读本知情同意书。

研究背景与研究目的：本研究的主要目的是调查加压素（20IU）或氯沙坦（50mg）对健康人群情绪和注意的调控作用。其中，加压素(Vasopressin)是人体自身会产生的一种神经肽，其分泌受到血管紧张素Ⅱ的调节；氯沙坦(Losartan)是一种血管紧张素 II 受体拮抗剂（ARB）。二者均被证实可以调节个体社会认知和情感加工,临床上应用于焦虑、应激障碍等的治疗，被认为在提高记忆力以及减少对负面情绪事件的记忆上具有良好效果。但目前还尚不清楚加压素和氯沙坦对社会-情绪信息的加工机制，因此本研究分为两个部分，实验 1 探讨加压素和氯沙坦对社会刺激（面孔）的注意特点，实验 2 探讨两种药物对非社会性刺激（动物）的注意特点。

如果您有以下情况之一，将不宜参与本研究：

神经精神疾病史。

心脏病史，包括心律失常、主动脉瓣狭窄或充血性心力衰竭；晕厥史或不明原因的意识丧失。

肝脏疾病史，包括胆汁淤积、胆道阻塞性疾病或严重肝功能障碍。

肾脏疾病史，包括肾结石或肾功能衰竭。

低钠血症（血钠<135mmol/L）或高钾血症（血钾＞5.5mmol/L）病史；糖尿病或尿崩症病史

已知对任何药物或激素过敏；对食物强烈的过敏反应史。

新冠或流感等感染，或不明原因发热。

高血压（血压≥140/90mmHg）或低血压（血压≤90/60mmHg）。

酗酒或吸毒史；其中吸烟（包括使用电子烟）是指≥10 支香烟或≥3 支雪茄或≥3 管/天。

献血（试验前 1 个月内）。

实验前 3 个月内服用口服避孕药或接受激素药物。

怀孕或哺乳。

实验流程

1、仔细阅读并在知情同意书上签字，随后在电脑上填写心理状况测评问卷（不超过 15 分钟）。

2、口服氯沙坦或安慰剂（1 分钟），随后等待 45 分钟。

3、口喷加压素或安慰剂（1 分钟）。随后等待 45 分钟。

4、进入眼动室完成视觉注意任务。（约 60 分钟）。

5、填写 SAI 问卷。

6、在实验前、服药后以及实验结束后各需测量一次血压。（合计 5 分钟）

关于药剂

实验中会出现的试剂有三种。

1.安慰剂喷雾和安慰剂胶囊。其中，安慰剂喷雾由水和甘油构成，安慰剂胶囊是维生素片，二者均不含任何药理成分，外形和真药一致。

2.加压素喷雾。加压素是一种人体荷尔蒙，通常由垂体分泌，并释放至血液或大脑中。在生理上，加压素可调节人体尿液中排出水的量，还会通过收缩血管来提高血压，从而调节人体的血压和体温。在临床领域，加压素与男性的利他行为，亲社会行为，以及婚姻满意度相关，和女性的伴侣关系的建立相关，因此被称作是社会性神经多肽。加压素常见不良反应有：心

跳加快、面部苍白、皮疹、恶心、呕吐等。本实验中使用的剂量微弱（20IU），在使用后约6.5小时后排出体外。

3. 氯沙坦胶囊（国药准字 HJ20171003）是一种血管紧张素 II 受体阻滞剂（ARB），在生理上主要用于治疗高血压和其他心血管疾病。在临床上，氯沙坦能有效改善个体对积极事件的记忆能力并在应激障碍、焦虑和抑郁等精神疾病的治疗上表现出潜在价值。作为一种可以长期服用的药物，氯沙坦和安慰剂之间只存在细小的、并且在安全范围内的副作用差异，例如乏力、疲劳、头晕、咳嗽等等。本实验单次使用的氯沙坦剂量为50mg，经验证对被试的血压几乎没有任何影响。该药物在服用后约12小时排出体外。

在实验当中，被试会随机分配到安慰剂喷雾+安慰剂胶囊、安慰剂喷雾+氯沙坦胶囊或加压素喷雾+安慰剂胶囊组。主试和被试均不知道将会分配到哪一组中。

关于口腔喷雾的使用方法：确保口腔清洁的前提下，摇匀瓶子，打开瓶盖，按下喷头使药剂喷出，舌上和舌下轮流各喷3次，共六次。每次喷后含30秒，不做吞咽动作，直至下次按压喷头。

隐私

所有收集到的数据和信息只用于研究。研究结果有可能会在科学杂志上出版或在科学会议上呈现，但您的名字或者其他个人信息将不会在任何发表的材料中被提及。

受试者之权利和义务

您参与研究是完全自愿的。在研究的任何时候，您都可以拒绝参与或退出研究。但如有不符合实验规定或威胁到您的实验安全的情况，研究者会结束您的实验：研究者相信这对您是最好的。同时，您有义务配合主试认真完成本实验，以保证实验质量。

参与本研究的可能获益：受试者可以通过本研究了解近期本人的心理状态（如焦虑、抑郁的程度），为自我积极调控心态提供参考依据。同时可以了解认知神经科学实验所涉及的一般流程，拓宽视野。另外受试者参与本实验会获得相应的报酬。

受试者申明：

我已经阅读了有关研究资料，所有的疑问都得到满意的答复，完全理解有关医学研究的资料以及该研究可能产生的风险和受益；确认已有充足的时间进行考虑；知晓参加研究是自愿的，有权在任何时间退出本研究，而不会受到歧视或报复，医疗待遇与权益不会受到影响；同意药品监督管理部门、伦理委员会或申办者查阅研究资料，签名已表示自愿参加研究。

签名：　　　　　　　日期：
联系方式：

研究者申明

我已经解释了本研究的目的、过程、可能的危险、潜在的获益、费用以及被试的权益等，并尽最大可能回答了与研究有关的问题。

签　名：[redacted]
联系方式：
日　期：

（被试）编号：____________

附件 2

除了计划书外实验计划可能存在的伦理问题说明

1. 实验风险描述，即未明确说明涉人实验是否存在任何风险，例如用于干预治疗的手段是什么，可能有什么风险；

实验风险描述和干预手段：

（1）实验中所涉及的设备无风险：采集眼动数据的设备，受试者仅需按照指导语完成相应的眼动任务，无任何风险。

（2）所使用的药物为加压素、氯沙坦钾片和安慰剂。加压素是人体本身会分泌的一种激素，研究表明其能够提高自信，改善孤独等。本研究针对健康成人（18-30 岁）使用 20 个国际单位(IU/单次）加压素，该剂量处于安全范围之内，可确保不会出现危害受试者健康的副作用。氯沙坦钾片是列入世界卫生组织基本药物目录，并经国家食品药品监督管理局（国药准字 H20000371）批准的，在全球广泛使用并且被认为是最安全，最有效的降压药物。与以往研究一致，本研究将使用 50mg 剂量氯沙坦，该剂量处于安全范围之内，可确保不会出现危害受试者健康的副作用。安慰剂，是不含任何药理成分的制剂或剂型，外形与真药相像，口喷安慰剂的成分为生理盐水和甘油，口服安慰剂则是维生素 C 片。

2. 涉人实验获益描述，即研究价值。特别地，如果存在风险，研究者应针对“风险/获益比”做明确说明；

（1）本研究结合行为指标和眼动技术探讨加压素和氯沙坦对个体社会认知及注意机制的影响。可以为以威胁性线索的过度关注和消极注意偏向为主要症状表现的精神疾病（如焦虑、抑郁、创伤后应激障碍等）提供新的药物治疗手段。

（2）受试者可通过本研究了解近期个人的心理状态（焦虑、抑郁的程度），同时可了解认知神经科学实验所涉及的一般流程，若受试者为本科生，这或将为其考取研究生时的专业选择提供参考。另受试者参与本研究会根据实验室被试费发放标准获得相应的报酬。

3. 如果涉人研究是基于数据二次使用(例如，数据已经存在或被收集，但研究目的不同)，说明“知情同意豁免”和“数据标识去除”问题；

无。

4. “本项目计划招募受试者人数/总人数”受试者人数明显偏低的合理性说明：

本项目计划招募受试者总人数 90 人。使用 G-Power 对本研究样本进行检验，招募人数在统计上满足了先验分析的样本量需求，不存在受试者人数偏低的情况。

附件 3

牵头单位伦理审查批件

电子科技大学为项目牵头单位

附件 4

培训时长证明

序号	姓名	单位	学号/工资号	培训起止时间	培训老师签名	认可积分
1	韩梦凡	生命科学与技术学院	202321140416	2024.3.1-2024.4.30	网站自学	[redacted]
总计分数						

附注：1.签名需要讲解专家 亲自签名；

2.相关证明材料

附件 5

项目计划书/申报书

研究背景

在人体内部存在着 RAAS 系统（肾素-血管紧张素-醛固酮）。RAAS 系统是调节血管功能、维持血压的重要的内分泌控制系统，也是人体血压、水和电解质平衡重要的调节系统。其中，血管紧张素Ⅱ是肾素作用血管紧张素原产生血管紧张素Ⅰ，再经转化酶的作用而生成。血管紧张素Ⅱ具有较高的生物活性，是最有效的加压物质。

研究发现，血管紧张素Ⅱ可能通过作用于神经垂体、SFO、OVLT 和视上核来增加血管加压素的释放。血管加压素（VP, vasopressin）也即抗利尿激素（ADH, Antidiuretic hormone），经由垂体后叶释放，在临床上被用来升高血压、止血等。在过去的十几年，越来越多的研究人员开始关注加压素对人类社会行为的调节作用。研究发现，大脑中的后叶加压素与男性的攻击性行为，利他行为，亲社会行为，以及婚姻满意度相关，和女性的伴侣关系的建立相关，被称作是社会性神经多肽。临床研究也表明，首发精神分裂症患者的脑脊液中的加压素的浓度要低于健康群体(Geng et al., 2017), 而持续服用加压素能够提高精神分裂症患者的记忆能力(Geng et al., 2017)以及加工情绪刺激的能力(Vadas et al., 2017)。

然而，以往关于加压素的研究主要采用鼻内给药的方式，因为神经肽如加压素可以通过嗅觉和三叉神经部分直接进入大脑并发挥作用（Yao et al., 2022），但越来越多的证据显示，加压素的功能作用也可以通过其在血液中的浓度增加而通过外周途径介导（Striepens et al., 2013; Xu et al., 2024）。这为通过口腔黏膜给药提供了新的可能性。虽然口喷加压素可能无法避免在经过胃肠道时的分解，但如果研究结果显示口喷与鼻喷加压素在治疗效果上具有相似性，这将强有力地支持外周给药途径的有效性。这意味着加压素不仅可以通过直接作用于大脑的方式发挥作用，也可以通过影响血液中的浓度而间接影响大脑功能。因此，本研究将通过口腔黏膜途径给药，我们实际上排除了药物直接通过嗅觉系统进入大脑的可能性，这使得我们能够更纯粹地评估外周给药途径的效果。通过将给药方式改为口喷，通过与之前鼻内给药的数据进行对比分析，这项研究将有助于理解口喷加压素是否能够保持与鼻喷相同的治疗效果，从而深入探讨其作用机制及临床应用前景。

氯沙坦（Losartan）是一种血管紧张素Ⅱ受体拮抗剂，主要通过阻断血管紧张素Ⅱ对 AT1 受体的结合来降低血压。在临床上用于治疗高血压和心血管疾病，此外还具有调节尿酸代谢的作用。研究发现，氯沙坦有助于调节个体对恐惧刺激的情绪反应(Reinecke et al., 2018)、促进恐惧情绪的消退以及对积极事件的学习和记忆(Pulcu et al., 2019; Zhou et al., 2019)。在 PTSD 等与恐惧情绪有关的精神疾病的治疗当中，氯沙坦表现出巨大的应用价值。氯沙坦和加压素一样对个体的社会功能和认知机制具有显著影响，然而，目前还没有研究探讨氯沙坦治疗后个体注意机制的变化以及对恐惧刺激的反应。考虑到二者功能和作用机制上的差异性，对自上而下和自下而上的注意控制的影响也可能存在不同。本研究将立足于前人研究的基础上，进一步探索被试在接受加压素或氯沙坦后其对个体注意控制行为的影响。

研究计划

本研究采用眼动技术记录被试的眼动数据，并结合行为数据探讨被试口喷加压素或口服催产素后期注意控制行为的变化以及对恐惧刺激的反应速度是否具有相似性或差异。在实验过程中被试需要完成的认知任务包括：眼动/反眼动任务、恐惧逼近任务。

本研究预计采集 90 名男性和女性被试的数据，被试被随机分到加压素组、氯沙坦组和安慰剂组，每组包含 15 名男性被试和 15 名女性被试。当被试来到实验室之后，受试者会填写

信息表和知情同意书，接下来进行20分钟左右的问卷填写（心境问卷，焦虑抑郁状态问卷等），然后按照相应要求使用加压素、氯沙坦或者安慰剂，正式实验前再次填写情绪问卷，随后开始练习实验和正式实验，任务结束后被试再次完成情绪问卷。

本研究数据分析分为眼动数据、反应时数据和问卷调查数据。通过对比分析实验组和对照组的问卷调查数据，探索加压素或氯沙坦对个体焦虑情绪等的影响；其次，通过反应时数据的差异探索加压素或催产素下个体对恐惧刺激检测能力的影响；最后，通过眼动数据分析探索加压素或氯沙坦对个体注意的影响，并探讨这种调节效应是否存在着性别差异。

主要参考文献

Bellaera, L., & von Mühlenen, A. (2019). Looming fear stimuli broadens attention in a local - global letter task. Progress in brain research, 247, 47-69.

Billington, J., Wilkie, R. M., Field, D. T., & Wann, J. P. (2011). Neural processing of imminent collision in humans. Proceedings of the Royal Society B: Biological Sciences, 278(1711), 1476-1481.

Nave, A. M., Tolin, D. F., & Stevens, M. C. (2012). Exposure therapy, D-cycloserine, and functional magnetic resonance imaging in patients with snake phobia: a randomized pilot study. The Journal of clinical psychiatry, 73(9), 7015.

Polák, J., Rádlová, S., Janovcová, M., Flegr, J., Landová, E., & Frynta, D. (2020). Scary and nasty beasts: Self - reported fear and disgust of common phobic animals. British Journal of Psychology, 111(2), 297-321.

Vagnoni, E., Lourenco, S. F., & Longo, M. R. (2012). Threat modulates perception of looming visual stimuli. Current biology, 22(19), R826-R827.

Vagnoni, E., Lourenco, S. F., & Longo, M. R. (2015). Threat modulates neural responses to looming visual stimuli. European Journal of Neuroscience, 42(5), 2190-2202.

Wright, J. W., & Harding, J. W. (2013). The brain renin - angiotensin system: a diversity of functions and implications for CNS diseases. Pflügers Archiv-European Journal of Physiology, 465, 133-151.

Xu, L., Ma, X., Zhao, W., Luo, L., Yao, S., & Kendrick, K. M. (2015). Oxytocin enhances attentional bias for neutral and positive expression faces in individuals with higher autistic traits. *Psychoneuroendocrinology*, *62*, 352–358. https://doi.org/10.1016/j.psyneuen.2015.09.002

Xu, T., Zhou, X., Jiao, G., Zeng, Y., Zhao, W., Li, J., ... & Becker, B. (2022). Angiotensin antagonist inhibits preferential negative memory encoding via decreasing hippocampus activation and its coupling with the amygdala. Biological Psychiatry: Cognitive Neuroscience and Neuroimaging, 7(10), 970-978.

Yao, S., Chen, Y., Zhuang, Q., Zhang, Y., Lan, C., Zhu, S., Becker, B., & Kendrick, K. M. (2023). Sniffing oxytocin: Nose to brain or nose to blood? *Molecular Psychiatry*. https://doi.org/10.1038/s41380-023-02075-2

Zhuang, Q., Zheng, X., Becker, B., Lei, W., Xu, X., & Kendrick, K. M. (2021). Intranasal vasopressin like oxytocin increases social attention by influencing top-down control, but additionally enhances bottom-up control. Psychoneuroendocrinology, 133, 105412. https://doi.org/10.1016/j.psyneuen.2021.105412

Zhou, F., Zhao, W., Qi, Z., Geng, Y., Yao, S., Kendrick, K. M., ... & Becker, B. (2021). A distributed fMRI-based signature for the subjective experience of fear. Nature communications, 12(1), 6643.

Kou, J., Lan, C., Zhang, Y., Wang, Q., Zhou, F., Zhao, Z., ... & Kendrick, K. M. (2021). In the nose or on the tongue?

Contrasting motivational effects of oral and intranasal oxytocin on arousal and reward during social processing. *Translational Psychiatry, 11*(1), 94.

Striepens, N., Kendrick, K. M., Hanking, V., Landgraf, R., Wüllner, U., Maier, W., & Hurlemann, R. (2013). Elevated cerebrospinal fluid and blood concentrations of oxytocin following its intranasal administration in humans. *Scientific reports, 3*(1), 3440.

Xu, D., Lan, C., Kou, J., Yao, S., Zhao, W., & Kendrick, K. M. (2024). Oromucosal Administration of Oxytocin: The Development of 'Oxipops'. *Pharmaceutics, 16*(3), 333.

Yao, S., & Kendrick, K. M. (2022). Effects of intranasal administration of oxytocin and vasopressin on social cognition and potential routes and mechanisms of action. *Pharmaceutics, 14*(2), 323.

University of Electronic Science and Technology of China
Research project ethics review application (Quick Review)

(Research involving human beings)

Project name: Effects of losartan and vasopressin on top-down and bottom-up control

Project leader: Professor Benjamin Becker

Applying School: School of Life Science and Technology

Research area: Cognitive Neuroscience

Application phase: Project application □ Paper publication☑

Date of application: 11 December 2023

Case number: 106142024050929462

Instructions for filling in the application form for ethical review of human research projects

(Continuously improve)

1. When submitting materials, the Research Project Ethics Review Application Form (hereinafter referred to as the "Application Form") must be truthfully filled in, and the corresponding boxes (, unless otherwise required, are mandatory;
2. Do not change the form format. Ensure the signature page does not span pages.
3. Involving relevant entity experiments, personal data privacy and other situations, the informed consent of the subject (signed by the subject) shall be submitted (Appendix 1)
4. A detailed research plan and a description of possible ethical issues (Appendix 2) must be submitted;
5. If our school is the participating unit, we need to provide the ethics review approval or situation statement (Appendix 3) of the leading unit;
6. After September 2021, researchers must provide either proof of relevant training duration (Appendix 4) or professional certifications. For projects not involving physical experiments, self-study materials on research ethics may be submitted.
7. The research proposal must be cleared for declassification before submitting the application form, project plan, and an overview of the research experiment content (Appendix 5).
8. If it involves the detection of biological samples abroad, the relevant government approval documents shall be provided;
9. If no attachments are required, enter "None".
10. The office of the Ethics Committee will collect the application form, organize an expert meeting for evaluation, and return the review results after evaluation;
11. The application materials shall be filled in the scientific research management system according to the application plan.

Research project ethics review application form

<table>
<tr><td colspan="3">Project name: Effects of losartan and vasopressin on top-down and bottom-up control</td></tr>
<tr><td colspan="2">Date of application: 11 December 2023</td><td>Subject: Human</td></tr>
<tr><td rowspan="3">Principal Investigator Information</td><td colspan="2">Project Leader: Professor Benjamin Becker, Germany</td></tr>
<tr><td colspan="2">Contact number: 15828121558 Email: ben_becker@gmx.de</td></tr>
<tr><td colspan="2">Secondary application unit: School of Life Science and Technology</td></tr>
<tr><td colspan="3">Briefly describe the purpose of the study and the research methods and contents carried out for the research object:
This study aims to investigate whether vasopressin (20 IU) and losartan (50 mg) affect attention control in the social-emotional anti-saccade paradigm and the threat looming task.
Vasopressin (a neuropeptide which can be stimulated by angiotensin II) and losartan (an angiotensin II type 1 receptor antagonist) are both known to modulate cognition and emotional processing. However, the precise mechanisms by which these hormones regulate attention allocation during threat-related stimuli—encompassing stimulus-driven bottom-up attention control and goal-directed top-down attention regulation—remain unclear.
This study aims to recruit 90 healthy college students enrolled in academic programs. Using a double-blind controlled clinical trial design, participants will be randomly assigned to three groups: vasopressin group, losartan group, and placebo group. Ninety minutes after treatment, subjects will first complete a social-emotional anti-saccade task, which employs social and non-social stimuli to investigate the specific effects of vasopressin or losartan on social and emotional processing. Subsequently, participants will complete a threat looming task using threat and non-threat stimuli to assess the medication's impact on attention regulation.
Potential ethical considerations: The study employs oral vasopressin, composed of vasopressin, normal saline, and glycerol. The potassium losartan tablets, approved by China's National Medical Products Administration (NMPA) (Approval No. H20000371), are globally recognized as the safest and most effective antihypertensive medication. All experimental equipment and procedures are safe, reliable, and non-toxic to humans. All personal data involved will be kept strictly confidential throughout the study.</td></tr>
<tr><td>Project source</td><td colspan="2">International cooperation project
National Fund Project (Foreign Outstanding Young Scholars Research Fund Project 32250610208))
Provincial and ministerial scientific research projects
Other</td></tr>
</table>

I promise	I hereby declare full responsibility for the authenticity of this experimental protocol. To address all safety risks associated with the project, I will strictly comply with national laws, regulations, and institutional guidelines, implement appropriate safety measures, and ensure all experiments are conducted under safe conditions. I voluntarily accept oversight from the Ethics Committee of the University of Electronic Science and Technology of China and the broader public.<br>Main Researcher: Han Mengfan, Ben Becker, Keith Kendrick
Review comments	Agree to the experiment plan.<br>Signature of the Director of the University Ethics Committee:

Note: Do not split this page across pages or change its format.

<table>
<tr><th colspan="5">I. Filling in research projects involving people</th></tr>
<tr><td colspan="5">The project plans to recruit 90 participants from a total population, with the trial expected to run from May 2023 to August 2024.</td></tr>
<tr><td colspan="2">The research process may cause serious harm to the subjects</td><td colspan="3">□ Yes ☑ No</td></tr>
<tr><td>Multicenter study</td><td colspan="4">☑ No
□ Yes: (International multi-center lead institution □ International multi-center participating institution
□ Domestic multi-center lead unit □ Domestic multi-center participating units
(If our school is the participating unit, we need to provide the approval of the leading unit's ethics review)</td></tr>
<tr><td>Whether it involves testing biological samples abroad</td><td colspan="4">☑ No
□Yes→ Sample type:
Surveillance project:
Name of laboratory:
Address:
Whether it involves testing of human genetic resources: □ No □ Yes</td></tr>
<tr><td>Research Type</td><td colspan="4">Interventional study☑ Non-interventional study □</td></tr>
<tr><td>subject investigated</td><td>Normal person ☑ Patient □</td><td>Participant age range</td><td colspan="2">18 to 30</td></tr>
<tr><td>vulnerable groupdisadvantaged group</td><td colspan="4">□ Mental illness □ Critical illness □ Pregnant women □ Illiterates □ Minors
Cognitive impairment □ Not involved ☑
Other:</td></tr>
<tr><td>Exclude object</td><td colspan="4">□ None □ Male □ Female ☑ Minor □ Other _______________</td></tr>
<tr><td>Special conditions required</td><td colspan="4">□Intensive Care □Surgery □Pediatric Intensive Care □Prosthetics
□ Intravenous infusion □ Gene therapy □ CT scan □ Gynecology
□Controlled substances (including narcotics and psychotropic drugs)
□ Quarantine zone □ Controlled drugs (narcotics/suppressive drugs)
□ Isolation zone ☑ No □ Other (specify)</td></tr>
</table>

	(1. Informed consent form and consent letter<br>(2. Possible ethical issues<br>(3. Approval document or explanation of the lead unit's ethics review<br>(4. Training duration certificate<br>(5. Complete version of the application or plan<br>(6. Other (check and attach on the back page)
Statement of Principal Investigator	Participants for this trial are enrolled adult university students recruited through online platforms. The recruitment process includes informed consent procedures and financial compensation, with strict protection of participant privacy. I hereby confirm the accuracy of the above information and assume full responsibility for quality assurance throughout the trial. I guarantee that all clinical trial data will be authentic, reliable, and conducted in compliance with ethical standards. The research team members have no conflicts of interest with this project. Any consequences arising from non-compliance will be borne solely by me.<br>Lead investigator: Han Mengfan, Ben Becker, Keith Kendrick<br>Date: 9 May 2024

Appendix 1

Informed consent form (for human subjects)

Dear Subject:
Hello everyone, we are researchers from the Key Laboratory of Neuroinformatics of the Ministry of Education at the University of Electronic Science and Technology of China. We welcome you to participate in this experiment. Please read this informed consent form carefully.

Research Background and Objectives: This study aims to investigate the effects of vasopressin (20 IU) or losartan (50 mg) on emotional regulation and attention in healthy individuals. Vasopressin, a neuropeptide naturally produced by the human body, is stimulated by angiotensin II, while losartan is an angiotensin II receptor antagonist (ARB). Both have been shown to modulate cognition and emotional processing, with clinical applications in treating anxiety and stress disorders. They are recognized for their effectiveness in improving memory retention and reducing negative emotional event recall. However, the mechanisms underlying vasopressin and losartan's impact on emotional information processing remain unclear. The study comprises two parts: Experiment 1 examines attentional characteristics when processing social stimuli (faces), while Experiment 2 investigates cognitive patterns during non-social stimuli (animals).

If you have any of the following conditions, you will not be eligible to participate in this study:
History of neuropsychiatric disorders.
Cardiac history, including arrhythmia, aortic stenosis or congestive heart failure; history of syncope or loss of consciousness of unknown cause.
History of liver disease, including cholestasis, obstructive biliary disease or severe liver dysfunction.
History of kidney disease, including kidney stones or renal failure.
History of hyponatremia (blood sodium $<$135mmol/L) or hyperkalemia (blood potassium $>$5.5mmol/L); history of diabetes or diabetes insipidus.
Known allergies to any medications or hormones; history of severe food allergies.
COVID-19 or flu infections, or fever of unknown origin.
Hypertension (blood pressure $\geq$140/90mmHg) or hypotension (blood pressure $\leq$90/60mmHg).
History of alcohol or drug abuse; smoking (including e-cigarettes) defined as $\geq$10 cigarettes or $\geq$3 cigars or $\geq$3 pipes/day.
Blood donation (within 1 month prior to the trial).
Use of oral contraceptives or hormone medications within 3 months prior to the experiment.
Pregnancy or breastfeeding.

Experimental procedure
1. Read carefully and sign the informed consent form, then fill in the psychological status assessment questionnaire (no more than 15 minutes).
2. Take losartan or a placebo orally (1 minute), then wait 45 minutes.
3. Spray vasopressin or placebo (1 minute). Wait 45 minutes.
4. Enter the eye movement room to complete the visual attention task (approximately 60 minutes).
5. Complete the SAI questionnaire.
6. Blood pressure should be measured once before the experiment, once after taking the medication,

and once after the experiment (total 5 minutes).

About the medication
There are three reagents that will be used in the experiment.
1. Placebo spray and placebo capsule. The placebo spray consists of water and glycerin, while the placebo capsule is a vitamin tablet. Both contain no active pharmaceutical ingredients and are indistinguishable from the actual medication in appearance.
2. Vasopressin Spray. Vasopressin, a human hormone typically secreted by the pituitary gland and released into the bloodstream or brain, plays a physiological role in regulating water excretion in urine and increasing blood pressure through vasoconstriction, thereby maintaining blood pressure and body temperature. In clinical research, vasopressin has been associated with altruistic behaviors, prosocial actions, and marital satisfaction in males, as well as relationship formation in females, earning it the designation of a "social neuropeptide". Common adverse reactions include tachycardia, facial pallor, rash, nausea, and vomiting. The dose used in this experiment (20 IU) was minimal.
3. Losartan capsules (China Drug Approval No. HJ20171003) are an angiotensin II receptor blocker (ARB) primarily used to treat hypertension and other cardiovascular diseases. Clinically, losartan has shown potential in improving memory for positive events and demonstrating therapeutic value in mental disorders such as stress-related disorders, anxiety, and depression. As a long-term medication, losartan exhibits minor side effects (e.g., fatigue, dizziness, and cough) that are within safe limits compared to placebo. In this study, a single 50mg dose of losartan had negligible impact on participants' blood pressure.

In the experiment, participants were randomly assigned to either placebo spray + placebo capsules, placebo spray + losartan capsules, or vasopressin spray + placebo capsules. Neither the experimenter nor the participants knew which group they would be assigned to.

For oral spray usage: After ensuring oral hygiene, shake the bottle well, open the cap, and press the nozzle to release the spray. Apply it to the tongue and under the tongue in alternating order, three times each, totaling six applications. After each spray, hold the spray in your mouth for 30 seconds without swallowing until the next press.

Conceal

All collected data and information are used solely for research purposes. While the findings may be published in scientific journals or presented at conferences, your name or other personal information will not be included in any published materials.

Rights and obligations of subjects

Your participation in the study is entirely voluntary. You may refuse to participate or withdraw from the study at any time. However, if there are circumstances that violate experimental protocols or pose risks to your safety, the researcher will terminate your participation when they deem it in your best interest. Additionally, you are obligated to fully cooperate with the principal investigator to ensure the quality of the study.

Potential benefits of participating in this study: Participants can assess their current psychological state (e.g., anxiety or depression levels) and gain insights for self-regulation. They will also learn about the general procedures of cognitive neuroscience experiments, broadening

their perspectives. Additionally, participants will receive appropriate compensation for their involvement.

Subject's declaration:

I have reviewed the study materials, received satisfactory answers to all questions, and fully understand the medical research information as well as the potential risks and benefits of this study. I confirm that I have sufficient time to consider my participation. I am aware that my involvement in this study is voluntary, and I have the right to withdraw at any time without facing discrimination or retaliation. My medical treatment and rights will not be affected. I agree to allow the drug regulatory authority, ethics committee, or sponsor to review the study materials. By signing, I confirm my voluntary participation in this study.

Signature: Date: Contact information:

Researcher's statement

I have explained the purpose, process, possible risks, potential benefits, costs and rights of the participants of this study, and tried my best to answer the questions related to the study.

Signature: Han Mengfan

(Subject) Number:___________

Contact:

Date:

Appendix 2

Explain the possible ethical issues of the experimental plan in addition to the plan

1.Experimental risk description, that is, it is not clearly stated whether there are any risks involved in the experiment, such as what is the means of intervention and treatment and what risks may be possible;

Experiment risk description and intervention measures:

(1)The equipment involved in the experiment is risk-free: the equipment for collecting eye movement data requires subjects to complete the corresponding eye movement tasks according to the instructions, and there is no risk.

(2)The medications used in this study were vasopressin, losartan and a placebo. Vasopressin, a hormone naturally produced by the human body, has been shown to enhance self-confidence and alleviate loneliness. This research administered 20 international units (IU) of vasopressin per dose to healthy adults aged 18-30, a dosage within the safe range to ensure no adverse health effects. Losartan potassium tablets, listed in the World Health Organization's Model List of Essential Medicines and approved by China's National Medical Products Administration (NMPA approval number H20000371), are globally recognized as the safest and most effective antihypertensive drugs. Consistent with previous studies, the 50mg dose of losartan was used, also within the safe range to prevent health risks. The placebo consisted of either saline solution and glycerin for oral inhalation or vitamin C tablets for oral administration.

2.Benefit description of human experimentation, i.e. research value. In particular, if there is a risk, the researcher should clearly explain the "risk/benefit ratio";

(1) This study investigates the effects of vasopressin and losartan on individual social cognition and attention mechanisms through behavioral indicators and eye-tracking techniques. The findings may provide novel pharmacological approaches for mental disorders characterized by excessive focus on threat-related cues and negative attentional bias, such as anxiety, depression, and post-traumatic stress disorder.

(2) Participants will gain insights into their recent psychological state (including anxiety and depression levels) through this study, while also understanding the general procedures of cognitive neuroscience experiments. For undergraduate participants, this experience may serve as a reference for their graduate school major selection. Additionally, participants will receive compensation according to the laboratory's standard participant fee structure.

3.If the research involves secondary use of data (e.g., data already exists or has been collected, but for a different purpose), address the issues of "informed consent exemption" and "data de-identification";

No

4.Justification for the significantly lower number of enrolled subjects compared to the total number of subjects in this project:

The study plans to enroll a total of 90 participants. Statistical analysis using G-Power confirmed that the recruitment size met the sample size requirements for the a priori analysis, with no under-enrollment.

Appendix 3

Ethical review approval of the lead unit

The University of Electronic Science and Technology of China is the lead unit of the project

Appendix 4

Training duration certificate

order number	surname and personal name	unit	Student ID/Employee ID	Training start and end times	Training instructor signature	Earn points
1	Han Mengfan	School of Life Science and Technology	202321140416	2023.11.20-2023.11.28	Website Self-Training	
Total Score						

Note: 1. The signature needs to be signed by the expert himself;

2. Relevant supporting materials

Appendix 5

Project Proposal/Statement

Research background

The human body contains the RAAS system (Renin-Angiotensin-Aldosterone), a crucial endocrine control system that regulates vascular function and maintains blood pressure. It also plays a vital role in regulating blood pressure and water-electrolyte balance [1,2]. Angiotensin II is produced when renin converts angiotensinogen into angiotensin I, which is then further processed by converting enzyme. With high biological activity, angiotensin II is recognized as the most potent vasoconstrictor [3,4].

Research indicates that angiotensin II may enhance vasopressin release by acting on the neurohypophysis, supraoptic nucleus (SFO), OVLT, and supraoptic nucleus. Vasopressin (VP), also known as antidiuretic hormone (ADH), is secreted by the posterior pituitary gland and clinically used for blood pressure elevation and hemostasis [5]. Over the past decade, increasing attention has been directed toward vasopressin's regulatory role in human social behavior. Studies reveal that posterior pituitary vasopressin correlates with male aggressive behavior, altruistic actions, prosocial conduct, and marital satisfaction, while in females, it is associated with partner formation. This neuropeptide is recognized as a key player in social regulation. Clinical data further demonstrate that initial schizophrenia patients exhibit lower vasopressin concentrations in cerebrospinal fluid compared to healthy individuals [6]. Notably, sustained vasopressin administration enhances both memory retention [6] and emotional stimulus processing capabilities in schizophrenia patients [7].

Losartan, an angiotensin II receptor antagonist, primarily lowers blood pressure by blocking angiotensin II from binding to AT1 receptors. Clinically used for hypertension and cardiovascular diseases, it also regulates uric acid metabolism. Research shows losartan helps modulate emotional responses to fear stimuli [8], promotes the fading of fear, and enhances learning and memory of positive events [9,10]. In treating fear-related mental disorders like PTSD, losartan demonstrates significant therapeutic value.

This study primarily investigates the effects of two medications on top-down and bottom-up attention control. Attention regulation is recognized as a critical factor in the development and maintenance of psychosocial disorders such as social anxiety and depression, which are closely associated with fear-related emotions. Previous research has demonstrated that vasopressin enhances bottom-up attention control while impairing top-down regulation. Individuals treated with vasopressin exhibit accelerated responses to emotionally charged faces and reduced inhibitory capacity for such reactions. Furthermore, vasopressin has shown anxiety-reducing effects [11].Given the functional and mechanistic differences between the two approaches, their impacts on top-down and bottom-up attention control may vary. Building on prior research, this study investigates how vasopressin or losartan affects participants' control behaviors.

Research report

This study employed eye-tracking technology to record participants' eye movement data, combined with behavioral data to examine whether their post-administration of oxytocin via intranasal spray or oral administration showed similar or differing patterns in attention control behavior and responses to fear stimuli. During the experiment, participants completed cognitive

tasks including anti-saccade tasks[12,13] and threat looming tasks [14–18].

This study plans to collect data from 90 male and female participants, who will be randomly assigned to three groups: vasopressin group, losartan group, and placebo group, with 15 male and 15 female participants in each group. Upon arrival at the laboratory, participants will complete an information form and informed consent form. They will then spend approximately 20 minutes completing questionnaires (including mood assessment, anxiety/depression status, etc.). Following this, they will receive vasopressin, losartan, or placebo as instructed. Before the formal experiment, participants will complete another mood questionnaire. The session will begin with practice trials and proceed to the main experiment. After completing the tasks, participants will take the mood questionnaire again.

This study's data analysis comprises three components: eye tracking data, reaction time data, and questionnaire survey data. First, by comparing questionnaire survey data between the experimental group and control group, we investigate the effects of vasopressin or losartan on individual anxiety levels. Second, through analyzing differences in reaction time data, we examine how vasopressin or oxytocin influences individuals' ability to detect fear stimuli. Finally, eye movement data analysis explores the impact of vasopressin or losartan on attention regulation, while investigating potential gender differences in these modulatory effects.

References

1. Nandave M. Introduction of renin-angiotensin-aldosterone system (RAAS). In: Nandave M, editor. Angiotensin-converting Enzyme Inhibitors vs Angiotensin Receptor Blockers: A Critical Analysis of Antihypertensive Strategies: A Machine-Generated Literature Overview. Singapore: Springer Nature; 2024. pp. 1–72. doi:10.1007/978-981-97-7380-0_1

2. Xu T, Fu K, Yao D, Ma C, Becker B. The brain renin angiotensin system: A novel precision target for neurofunctional symptom regulation. OSF; 2025. doi:10.31234/osf.io/fbcv4_v1

3. Nehme A, Zouein FA, Deris Zayeri Z, Zibara K. An update on the tissue renin angiotensin system and its role in physiology and pathology. J Cardiovasc Dev Dis. 2019;6: 14. doi:10.3390/jcdd6020014

4. Wright JW, Harding JW. The brain renin–angiotensin system: A diversity of functions and implications for CNS diseases. Pflugers Arch - Eur J Physiol. 2013;465: 133–151. doi:10.1007/s00424-012-1102-2

5. Yao S, Chen Y, Zhuang Q, Zhang Y, Lan C, Zhu S, et al. Sniffing oxytocin: Nose to brain or nose to blood? Mol Psychiatry. 2023;28: 3083–3091. doi:10.1038/s41380-023-02075-2

6. Geng C-H, Wang C, Yang J, Wang H, Ma R-Q, Liu X, et al. Arginine vasopressin improves the memory deficits in han Chinese patients with first-episode schizophrenia. Peptides. 2017;97: 8–15. doi:10.1016/j.peptides.2017.09.002

7. Vadas L, Bloch B, Levin R, Shalev I, Israel S, Uzefovsky F, et al. Sex-specific effect of intranasal vasopressin, but not oxytocin, on emotional recognition and perception in schizophrenia patients. European Psychiatry. 2017;41: S387–S388. doi:10.1016/j.eurpsy.2017.02.430

8. Reinecke A, Browning M, Klein Breteler J, Kappelmann N, Ressler KJ, Harmer CJ, et al. Angiotensin

regulation of amygdala response to threat in high-trait-anxiety individuals. Biol Psychiatry Cogn Neurosci Neuroimaging. 2018;3: 826–835. doi:10.1016/j.bpsc.2018.05.007

9. Pulcu E, Shkreli L, Holst CG, Woud ML, Craske MG, Browning M, et al. The effects of the angiotensin II receptor antagonist losartan on appetitive versus aversive learning: A randomized controlled trial. Biol Psychiatry. 2019;86: 397–404. doi:10.1016/j.biopsych.2019.04.010

10. Zhou F, Geng Y, Xin F, Li J, Feng P, Liu C, et al. Human extinction learning is accelerated by an angiotensin antagonist via ventromedial prefrontal cortex and its connections with basolateral amygdala. Biol Psychiatry. 2019;86: 910–920. doi:10.1016/j.biopsych.2019.07.007

11. Zhuang Q, Zheng X, Becker B, Lei W, Xu X, Kendrick KM. Intranasal vasopressin like oxytocin increases social attention by influencing top-down control, but additionally enhances bottom-up control. Psychoneuroendocrinology. 2021;133: 105412. doi:10.1016/j.psyneuen.2021.105412

12. Zhuang Q, Zheng X, Yao S, Zhao W, Becker B, Xu X, et al. Oral administration of oxytocin, like intranasal administration, decreases top-down social attention. Int J Neuropsychopharmacol. 2022;25: 912–923. doi:10.1093/ijnp/pyac059

13. Xu X, Li J, Chen Z, Kendrick KM, Becker B. Oxytocin reduces top-down control of attention by increasing bottom-up attention allocation to social but not non-social stimuli - a randomized controlled trial. Psychoneuroendocrinology. 2019;108: 62–69. doi:10.1016/j.psyneuen.2019.06.004

14. Bellaera L, von Mühlenen A. Looming fear stimuli broadens attention in a local-global letter task. Prog Brain Res. 2019;247: 47–69. doi:10.1016/bs.pbr.2019.03.020

15. Vagnoni E, Lourenco SF, Longo MR. Threat modulates perception of looming visual stimuli. Current Biology. 2012;22: R826–R827. doi:10.1016/j.cub.2012.07.053

16. Vagnoni E, Lourenco SF, Longo MR. Threat modulates neural responses to looming visual stimuli. Eur J Neurosci. 2015;42: 2190–2202. doi:10.1111/ejn.12998

17. Billington J, Wilkie RM, Field DT, Wann JP. Neural processing of imminent collision in humans. Proc Biol Sci. 2011;278: 1476–1481. doi:10.1098/rspb.2010.1895

18. Nave AM, Tolin DF, Stevens MC. Exposure therapy, D-cycloserine, and functional magnetic resonance imaging in patients with snake phobia: A randomized pilot study. J Clin Psychiatry. 2012;73: 1179–1186. doi:10.4088/JCP.11m07564
